# Physiological medium and 3-hydroxybutyrate modulate autophagy-linked organelle remodeling in human external urethral sphincter myoblasts

**DOI:** 10.1038/s41598-026-43453-4

**Published:** 2026-03-16

**Authors:** Hironori Kai, Shinro Hata, Noriko Hamamatsu, Ryoko Shiraishi, Toshitaka Shin

**Affiliations:** Department of Urology, Faculty of Medicine, Oita University, Yufu, 879-5593 Oita Japan

**Keywords:** Autophagy, stress urinary incontinence, external urethral sphincter, 3-hydroxybutyrate, physiological medium, Biochemistry, Cell biology, Physiology

## Abstract

Autophagy-linked organelle remodeling is essential for skeletal muscle differentiation and is closely linked to the metabolic environment. The ketone body 3-hydroxybutyrate (3HB) serves as an alternative energy substrate and signaling molecule that modulates organelle function and myogenic programs. This study investigated how extracellular metabolic conditions and 3HB regulate autophagy-linked organelle remodeling in human external urethral sphincter (hEUS) myoblasts. Immortalized hEUS myoblasts (US2-KD) were differentiated under four conditions combining high-glucose Dulbecco’s modified Eagle’s medium (HG-DMEM) or low-glucose physiologically formulated minimum essential medium (LG-MEM) with or without 3HB. Metabolomic profiling revealed that the medium composition predominantly shaped energy and amino acid pathways, whereas 3HB induced subtler, context-dependent shifts in metabolites related to autophagy and mitochondrial function. At the cellular level, LG-MEM accelerated myogenic differentiation compared to HG-DMEM, with earlier induction of *MYOG* and *MYH7* and faster maturation of myotubes. LG-MEM also altered LC3B expression patterns, while transmission electron microscopy showed fewer excess autophagosomes and autolysosomes along with more prominent myofibril-like ultrastructure, consistent with more efficient autophagic activity and organelle remodeling. These findings suggest that a physiological metabolic environment facilitates autophagy-linked organelle remodeling in hEUS myoblasts and that 3HB acts as a fine-tuning signal, potentially supporting metabolism-oriented approaches for stress urinary incontinence.

**Supplementary Information:**

The online version contains supplementary material available at 10.1038/s41598-026-43453-4.

## Introduction

Urinary leakage during everyday activities such as coughing, sneezing, physical exertion, or lifting markedly impairs quality of life and represents a major component of lower urinary tract dysfunction. Stress urinary incontinence (SUI), defined as involuntary leakage associated with increased abdominal pressure, typically occurs in women in association with aging, childbirth, or menopause and in men after radical prostatectomy, and is further promoted by obesity and diabetes^1–5^. ​SUI limits daily activities such as walking, housework, and sports, and also discourages social participation, leading to social isolation and deterioration of mental health; in aging societies with a growing burden of lifestyle-related diseases, it has therefore become a significant societal concern. In this context, regenerative approaches that directly target urethral sphincter regeneration and functional recovery—such as platelet-rich plasma (PRP) injection, stem cell transplantation, and stem cell-derived secretomes—have attracted increasing attention, although their efficacy, safety, and long-term reproducibility still vary according to cell type, patient background, and administration protocols^6–9^. Thus, there remains a need for basic knowledge that can support the development of more reliable regenerative strategies tailored to individual pathophysiology.​ Our previous studies established a primary culture system for human external urethral sphincter (hEUS) satellite cells and clarified the roles of differentiation-regulating factors such as IGF-1 ^10^, HGF^10,11^, and TNF-α^13^. We also reported that, compared with the rectus abdominis, the external urethral sphincter of patients with bladder cancer contains a higher proportion of abnormal mitochondria^12^, that the tricarboxylic acid (TCA) cycle acts as a metabolic hub for differentiation in hEUS cells, and that inhibition of TCA cycle activity markedly suppresses hEUS differentiation^13^.

Recent studies have shown that ketone bodies, particularly 3-hydroxybutyrate (3HB), exert multiple physiological effects, including stimulation of myogenic differentiation, enhancement of mitochondrial function, and attenuation of muscle atrophy, while activation of autophagy, which maintains intracellular homeostasis, has emerged as a promising therapeutic target for tissue regeneration and differentiation^14–17^. Based on these insights, we hypothesized that 3HB would promote differentiation of hEUS myoblasts. In initial experiments using high-glucose Dulbecco’s modified Eagle’s medium (4.5 g/L; HG-DMEM), however, 3HB produced only minimal effects on myogenic differentiation. To better approximate physiological conditions and reduce confounding compensatory effects of amino acids, we next examined 3HB in low-glucose minimum essential medium (1 g/L; LG-MEM), whose amino acid and nutrient composition is closer to the physiological milieu. Strikingly, switching the culture medium from HG-DMEM to LG-MEM alone enhanced differentiation more strongly than 3HB supplementation, indicating that the impact of 3HB is highly dependent on the metabolic environment of the cells. To dissect the main and interactive effects of glucose concentration and 3HB, we therefore designed a four-group comparison: HG-DMEM, HG-DMEM + 3HB, LG-MEM, and LG-MEM + 3HB.​.

In this study, hEUS myoblasts cultured under four defined conditions were analyzed using an integrated approach that combined metabolomics, gene expression, and transmission electron microscopy–based assessment of autophagic activity and organelle morphology. Rather than relying solely on PRP or cell therapy^6–9^, we focused on metabolic modulation, molecular targeting, and autophagy activation to establish reproducible models that can be adapted to specific hEUS pathologies. These findings are expected to provide fundamental insights into urethral sphincter cell biology and metabolism and to help guide future regenerative strategies for SUI and other lower urinary tract dysfunctions.

## Results

As described in the Materials and Methods, phase-contrast images and metabolomics/qRT-PCR data were obtained from the same time-course batch of US2-KD cultures, whereas all other assays were performed in independent batches of US2-KD cells treated under the same conditions.

**Fig. 1:**
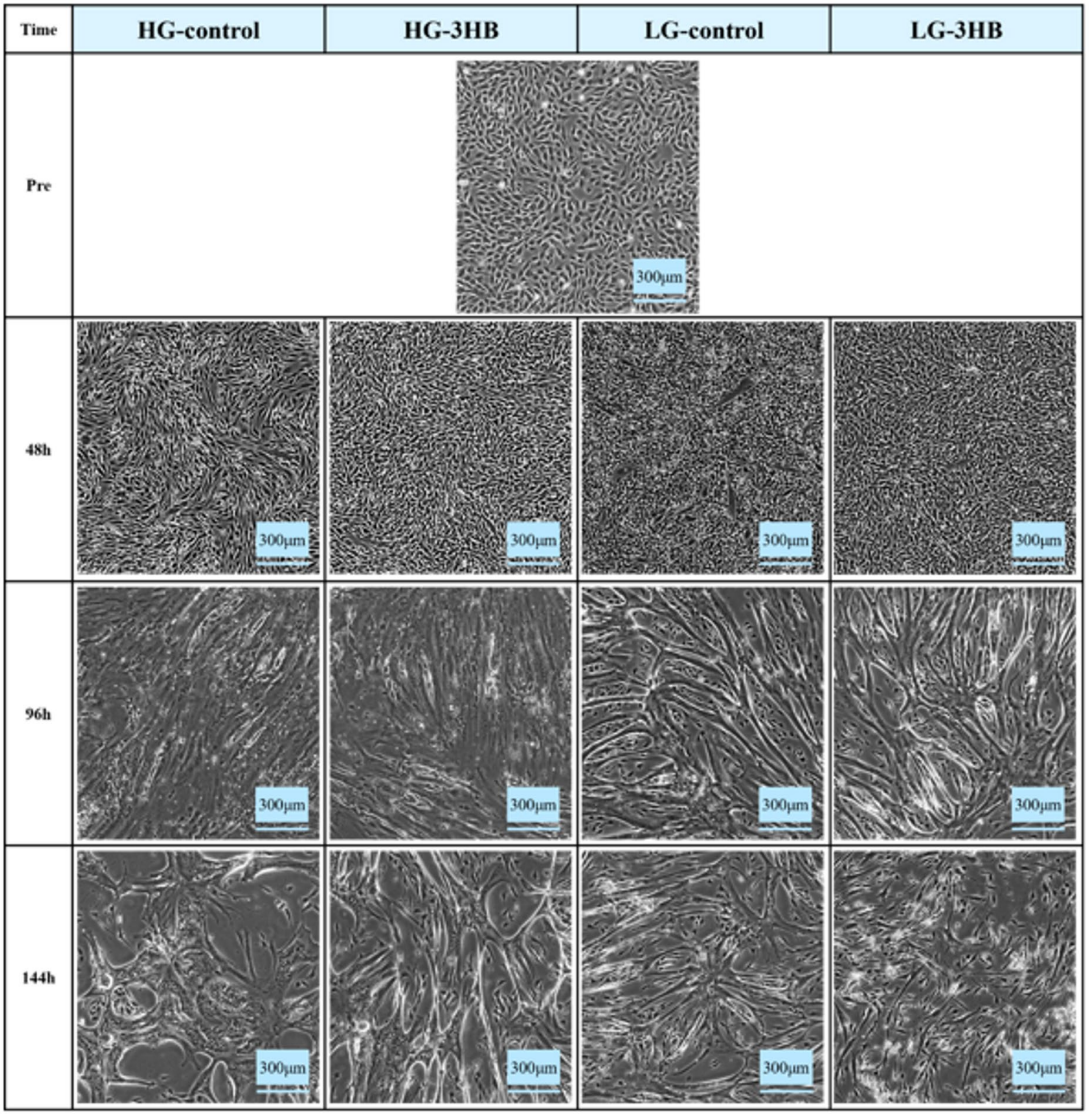
Phase-contrast images of myogenic differentiation under different culture conditions. Representative phase-contrast micrographs of US2-KD cells at 96 and 144 h after differentiation induction. Cells were cultured in high-glucose DMEM (HG-control), HG-DMEM with 3-hydroxybutyrate (HG-3HB), low-glucose MEM (LG-control), or LG-MEM with 3-hydroxybutyrate (LG-3HB). The images demonstrate the morphological changes and myotube formation observed under each condition at each time point. Scale bar: 300 μm (original magnification ×40).

**Fig. 2:**
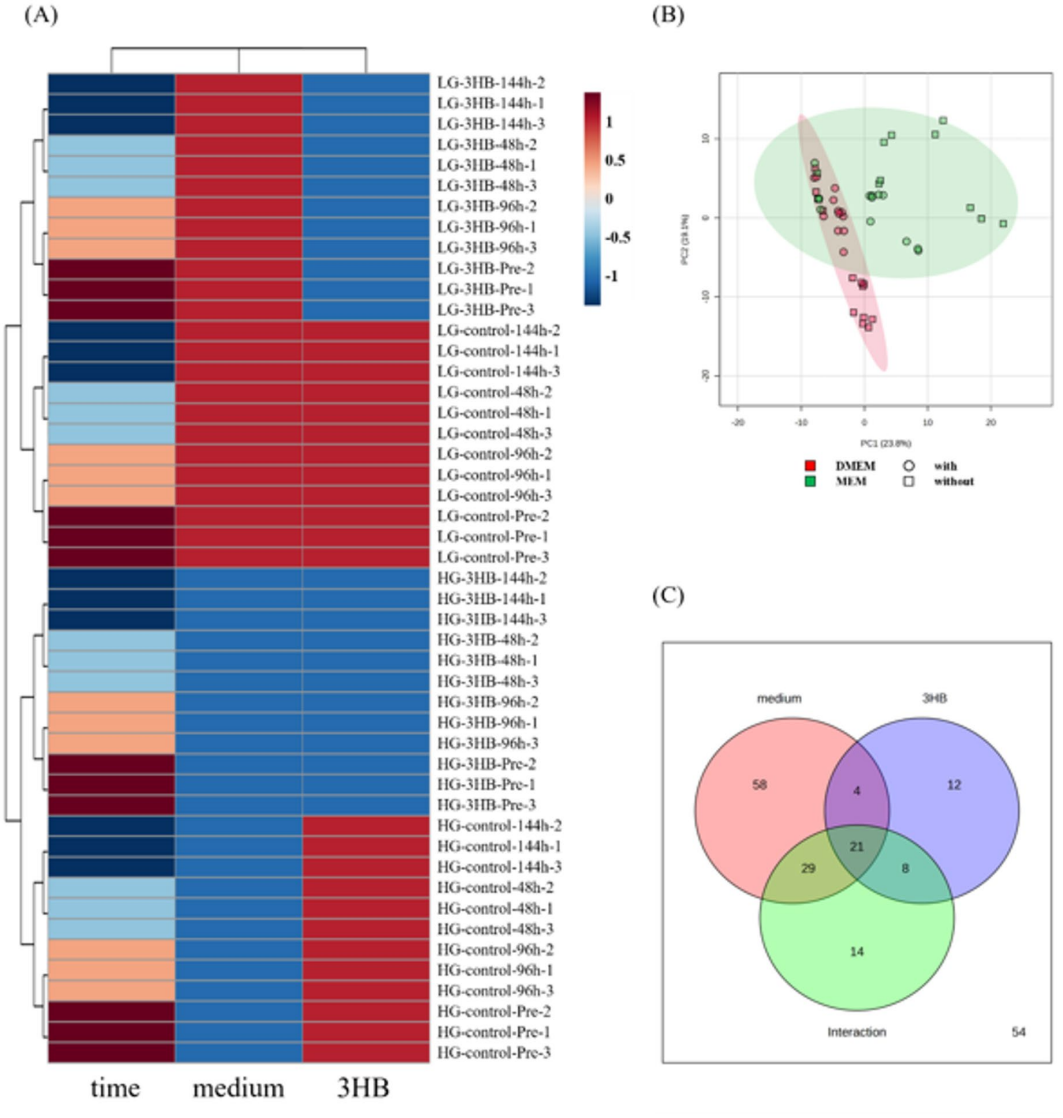
Metabolomic profiling distinguishes effects of medium and 3-hydroxybutyrate during myogenic differentiation. (**A**) Clustered heatmap of metabolomic profiles. Unsupervised hierarchical clustering of metabolite abundance for all sample groups at each time point (Pre, 48 h, 96 h, 144 h) under high-glucose DMEM (HG-control, HG-3HB) and low-glucose MEM (LG-control, LG-3HB) conditions. The clustering in Fig. 2A summarizes, at the sample level, the effects of time, medium, and 3HB, rather than displaying patterns of all individual metabolites. A global heatmap displaying all detected metabolites across all samples is provided as Supplementary Fig. S1. (**B**) Principal component analysis (PCA) plot of the samples. Metabolomic variance was visualized using PCA; ellipses represent sample distributions for DMEM and MEM with or without 3-hydroxybutyrate. Red and green indicate DMEM and MEM, respectively, and circles and squares indicate cultures with and without 3-hydroxybutyrate, respectively.　(C) Venn diagram of metabolites with significant effects. Numbers indicate metabolites showing significant main effects of medium, 3-hydroxybutyrate (3HB), or their interaction, as determined by two-way ANOVA. Interaction denotes metabolites showing a significant medium × 3-hydroxybutyrate interaction term in the two-way ANOVA. 3HB, 3-hydroxybutyrate; DMEM, Dulbecco’s modified Eagle’s medium; MEM, minimum essential medium; HG, high-glucose; LG, low-glucose. Metabolomic profiling was performed on *n* = 3 biological replicates for each condition.

US2-KD cells were examined using phase-contrast microscopy (Fig. 1). Under HG conditions, myotube formation progressed gradually, and well-formed myotube structures became apparent at 144 h. No clear morphological differences were observed between HG-control and HG-3HB. In contrast, extensive myotube formation was evident at 96 h in the LG groups. At 144 h, many of the myotubes that had formed in LG-3HB were lost, whereas in LG-control myotubes were re-formed from the remaining myoblasts after detachment, giving the impression of secondary differentiation. To explore the metabolic basis for these differences in differentiation dynamics induced by medium composition and 3HB, we first performed metabolomic analysis of the culture supernatants. Figure 2 shows the results of the statistical metadata analysis of the metabolomics data from the culture supernatants. The cluster heatmap (Fig. 2A) demonstrates that the metabolomic profiles were clearly separated by medium conditions compared to the 3HB condition. A global heatmap displaying all detected extracellular metabolites across all samples is provided as Supplementary Fig. S1. In the principal component analysis (Fig. 2B), PC1 and PC2 accounted for 23.8% and 19.1% of the total variances, respectively. The Venn diagram (Fig. 2C) summarizes the metabolites identified using two-way ANOVA. Twelve metabolites were significantly affected by 3HB, 58 by the medium, and 14 by their interaction, indicating that the medium had the most significant impact on the metabolite composition.

Supplementary Tables S1–4 summarizes the metabolic pathways that were significantly enriched in each group at each time point, together with their FDR and pathway impact values. At Pre–48 h, all four groups shared a similar early differentiation profile dominated by core energy metabolism. From 48 to 96 h, HG-control was mainly associated with taurine/hypotaurine and other amino acid–related pathways, whereas HG-3HB showed prominent enrichment of sulfur-containing amino acid and antioxidant pathways. In LG-control, amino acid- and cofactor-related pathways were predominant, while LG-3HB exhibited relatively higher enrichment of carbohydrate- and glycerolipid-related pathways. At 96–144 h, HG groups were characterized by taurine/hypotaurine, sulfur-containing amino acid, sphingolipid, and other amino acid/organic acid pathways, including nucleotide metabolism in HG-3HB, whereas both LG groups consistently showed strong enrichment of amino acid and organic acid/energy metabolism accompanied by selected carbohydrate- and nucleotide-related pathways.

**Fig. 3:**
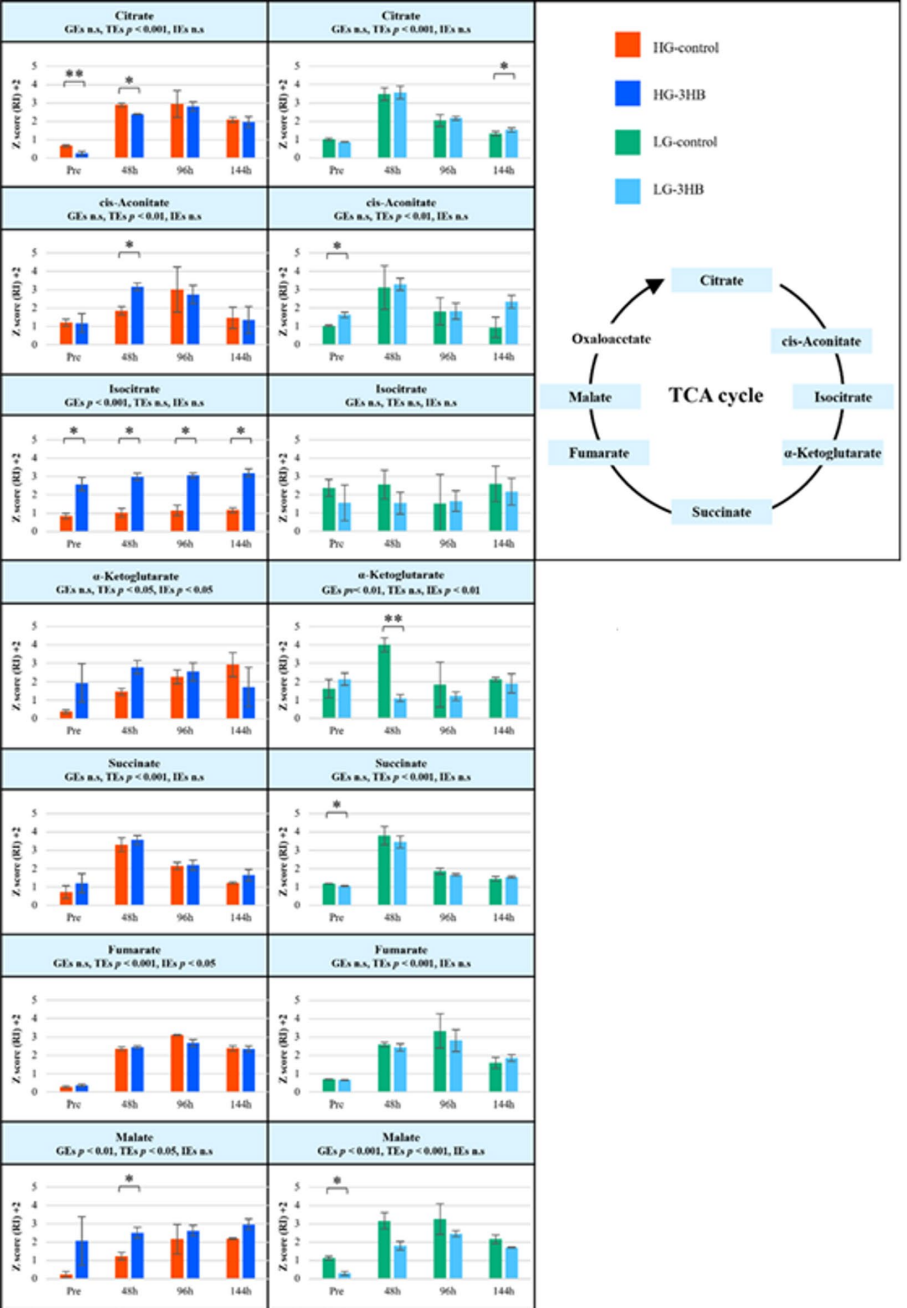
Z-score analysis of TCA cycle intermediates during myogenic differentiation under different culture conditions. Levels of key TCA cycle metabolites (citrate, cis-aconate, isocitrate, α-ketoglutarate, succinate, fumarate, and malate) are presented as Z-scores for the high-glucose control, HG-3HB, low-glucose control, and LG-3HB at each time point (Pre, 48 h, 96 h, and 144 h). The right panel illustrates the positions of each measured metabolite in the TCA cycle. Significant differences are indicated by *p < 0.05 and **p < 0.01. TCA, tricarboxylic acid; h, hours; GEs, group effects; TEs, time effects; IEs, interaction effects.

Next, we focused on energy-related metabolites and signaling-associated metabolites that have been reported to be targets of 3HB. With respect to energy metabolism, we examined the TCA cycle as a central metabolic hub and performed a detailed analysis of the TCA cycle intermediates. Figure 3 illustrates the intergroup differences in the TCA cycle intermediates. In HG group, the addition of 3HB increased the levels of several TCA cycle intermediates, suggesting that cellular energy metabolism via the TCA cycle was activated. In contrast, in the LG group, 3HB administration was associated with decreased TCA cycle intermediate levels, suggesting possible suppression of TCA cycle activity.

Following the analysis of energy-related metabolites, we focused on metabolites associated with 3HB signaling that are linked to autophagy, mitochondrial function, and epigenetic regulation. The effects of 3HB in the HG and LG groups are summarized in Table 1. Autophagy-related metabolites showed significant changes in catechol, dopamine, and serotonin levels in the HG group and in oleamide, catechol, dopamine, and p-octopamine levels in the LG group. The mitochondria-related metabolites that showed significant changes in the HG group included 2-methyl-3-hydroxybutyric acid, taurine, hypotaurine, methylmalonic acid, hypoxanthine, and ribonolactone, while those in the LG group included 2-methyl-3-hydroxybutyric acid, 3-hydroxyisobutyric acid (3-HIB), taurine, and ribonolactone in the LG group. For epigenetic regulation–related metabolites, hydroxyphenyllactic acid and phenyllactic acid showed significant differences in both the HG and LG groups. These results indicate that in both HG and LG conditions, 3HB and time-course exposure are accompanied by the remodeling of multiple signaling-related metabolic pathways.

**Table 1:** Statistical analysis of key metabolites related to autophagy, mitochondria, and epigenetic regulation. Summary of metabolites showing significant changes in group, time, or interaction effects based on paired ANOVA. Metabolites were grouped by functional category (autophagy-related, mitochondria-related, epigenetic regulators/other), and the results are presented for the HG-control/HG-3HB and LG-control/LG-3HB conditions. Statistical significance is indicated for each ANOVA factor (group, time, interaction); n.s. not significant.

Category	Metabolites	ANOVA	HG-control/HG-3HB	LG-control/LG-3HB
**Autophagy-related metabolites**	Oleamide	Group	n.s.	*p* < 0.01
		Time	n.s.	n.s.
		Interaction	n.s.	n.s.
	**Catechol**	Group	*p* < 0.001	*p* < 0.05
		Time	*p* < 0.001	*p* < 0.01
		Interaction	*p* < 0.001	n.s.
	**Dopamine**	Group	*p* < 0.05	*p* < 0.001
		Time	*p* < 0.05	*p* < 0.001
		Interaction	n.s.	*p* < 0.05
	**Serotonin**	Sample	*p* < 0.001	n.s.
		Column.s.	*p* < 0.01	*p* < 0.01
		Interaction	*p* < 0.05	n.s.
	**p-Octopamine**	Group	n.s.	*p* < 0.05
		Time	n.s.	*p* < 0.01
		Interaction	n.s.	n.s.
**Mitochondria-related metabolites**	**2-Methyl-3-hydroxybutyric acid**	Group	*p* < 0.001	*p* < 0.001
		Time	*p* < 0.05	n.s.
		Interaction	*p* < 0.05	n.s.
	**(S)-3-hydroxyisobutyric acid**	Group	n.s.	*p* < 0.001
		Time	*p* < 0.01	*p* < 0.001
		Interaction	*p* < 0.01	n.s.
	**Taurine**	Group	*p* < 0.01	*p* < 0.05
		Time	*p* < 0.001	*p* < 0.001
		Interaction	n.s.	n.s.
	**Hypotaurine**	Group	*p* < 0.05	n.s.
		Time	*p* < 0.001	*p* < 0.01
		Interaction	*p* < 0.05	n.s.
	**Methylmalonic acid**	Group	*p* < 0.05	n.s.
		Time	n.s.	*p* < 0.001
		Interaction	n.s.	n.s.
	**Hypoxanthine**	Group	*p* < 0.05	n.s.
		Time	*p* < 0.001	*p* < 0.01
		Interaction	*p* < 0.05	n.s.
	**Ribonolactone**	Group	*p* < 0.01	*p* < 0.05
		Time	*p* < 0.01	*p* < 0.01
		Interaction	*p* < 0.05	*p* < 0.01
**Epigenetic regulators / Other metabolites**	**Phenylpyruvic acid**	Group	*p* < 0.001	*p* < 0.001
		Time	n.s.	n.s.
		Interaction	n.s.	n.s.
	**Phenyllactic acid**	Group	*p* < 0.001	*p* < 0.001
		Time	*p* < 0.01	*p* < 0.001
		Interaction	n.s.	*p* < 0.01
	**Hydroxyphenyllactic acid**	Group	n.s.	*p* < 0.001
		Time	n.s.	n.s.
		Interaction	n.s.	n.s.
	**4-Hydroxyphenylpyruvic acid**	Group	n.s.	n.s.
		Time	n.s.	n.s.
		Interaction	*p* < 0.05	n.s.

**Fig. 4:**
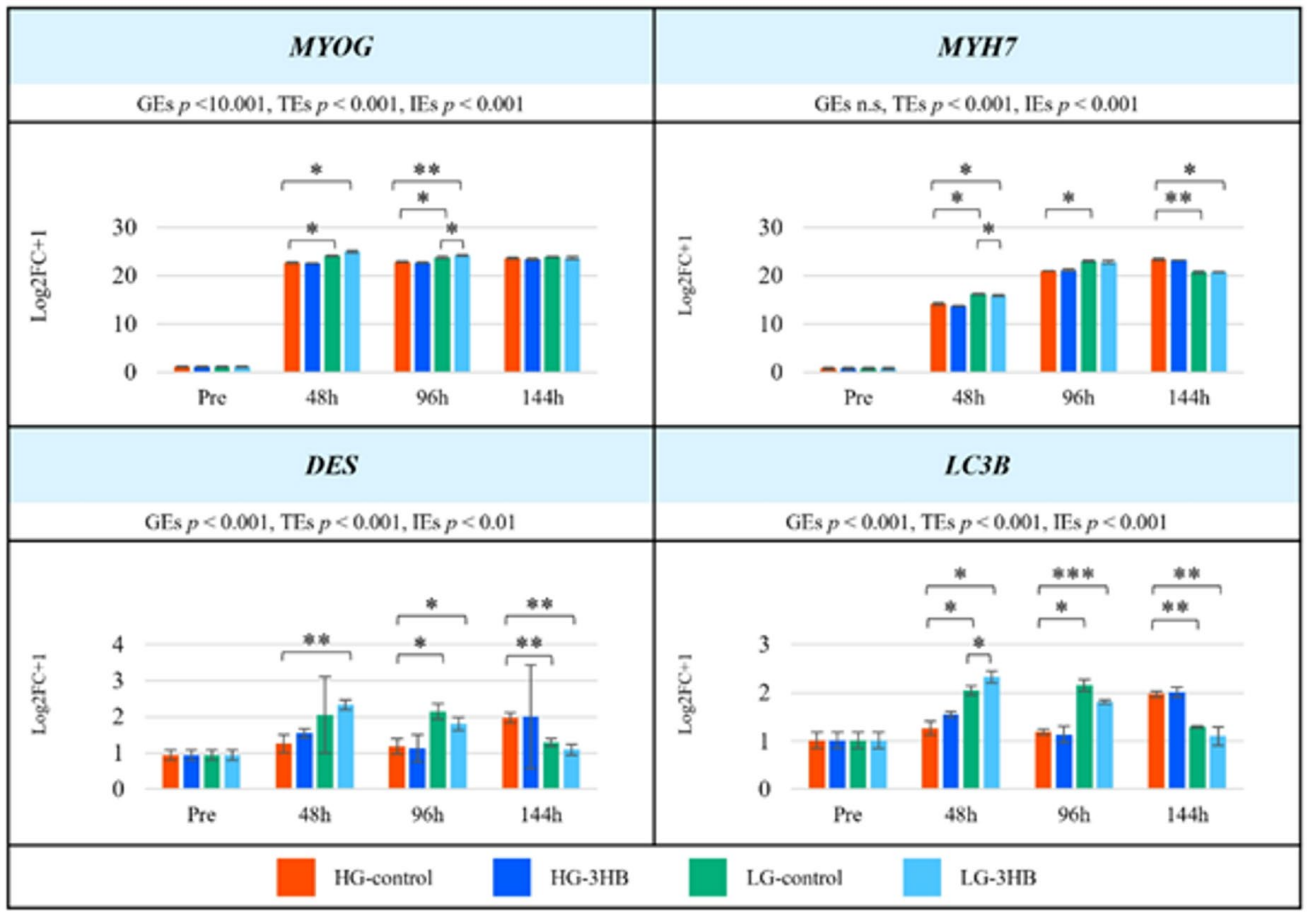
Temporal expression patterns of myogenic and autophagy-related genes under different culture conditions. Expression levels of myogenic markers (*MYOG*, *MYH7*, and *DES*) and the autophagy-related gene (*LC3B*) in US2-KD cells. Gene expression was assessed at pre-differentiation, 48 h, 96 h, and 144 h under four conditions: high-glucose control (HG-control), high-glucose DMEM with 3-hydroxybutyrate (HG-3HB), low-glucose control (LG-control), and low-glucose MEM with 3-hydroxybutyrate (LG-3HB) (*n* = 3 per group). Significant differences are indicated by *p < 0.05, **p < 0.01, and ***p < 0.001. Error bars represent standard deviations. Data are shown as log2FC relative to the control, with statistical results indicated for GEs, TEs, and IEs. DMEM, Dulbecco’s modified Eagle’s medium; MEM, minimum essential medium; 3HB, 3-hydroxybutyrate; GEs, group effects; TEs, time effects; IEs, interaction effects; h, hours.

We then examined the changes in myogenic and autophagy-related gene expression (Fig. 4). MYOG increased after 48 h in all groups and was significantly higher in LG-control than in HG-control at 48–96 h, but this difference disappeared by 144 h (*p* < 0.05). In addition, 3HB treatment significantly increased *MYOG* expression in LG-3HB compared with LG-control at 96 h (*p* < 0.05). *MYH7* was already markedly upregulated in LG-control compared with HG-control at 48 and 96 h. In contrast, *MYH7* expression in HG-control continued to rise and reached its highest level at 144 h. With respect to 3HB, a significant difference was observed between LG-control and LG-3HB at 48 h (*p* < 0.05). *DES* showed a pattern similar to that of *MYH7*: its expression tended to be higher in LG-control and LG-3HB at 96 h, but declined in both LG groups by 144 h, while increasing in HG-control and HG-3HB at the same time point. *LC3B*, an indicator of autophagy induction, was significantly upregulated in LG-control at 48 and 96 h (*p* < 0.05), whereas in HG-control and HG-3HB it peaked later at 144 h. A significant difference between LG-control and LG-3HB was also detected at 48 h. Two-way ANOVA revealed significant group effects, time effects, and interaction effects for all four genes (*MYOG*,* MYH7*,* DES*, and *LC3B*; GEs *p* < 0.001, TEs *p* < 0.001, IEs *p* < 0.001).

**Fig. 5:**
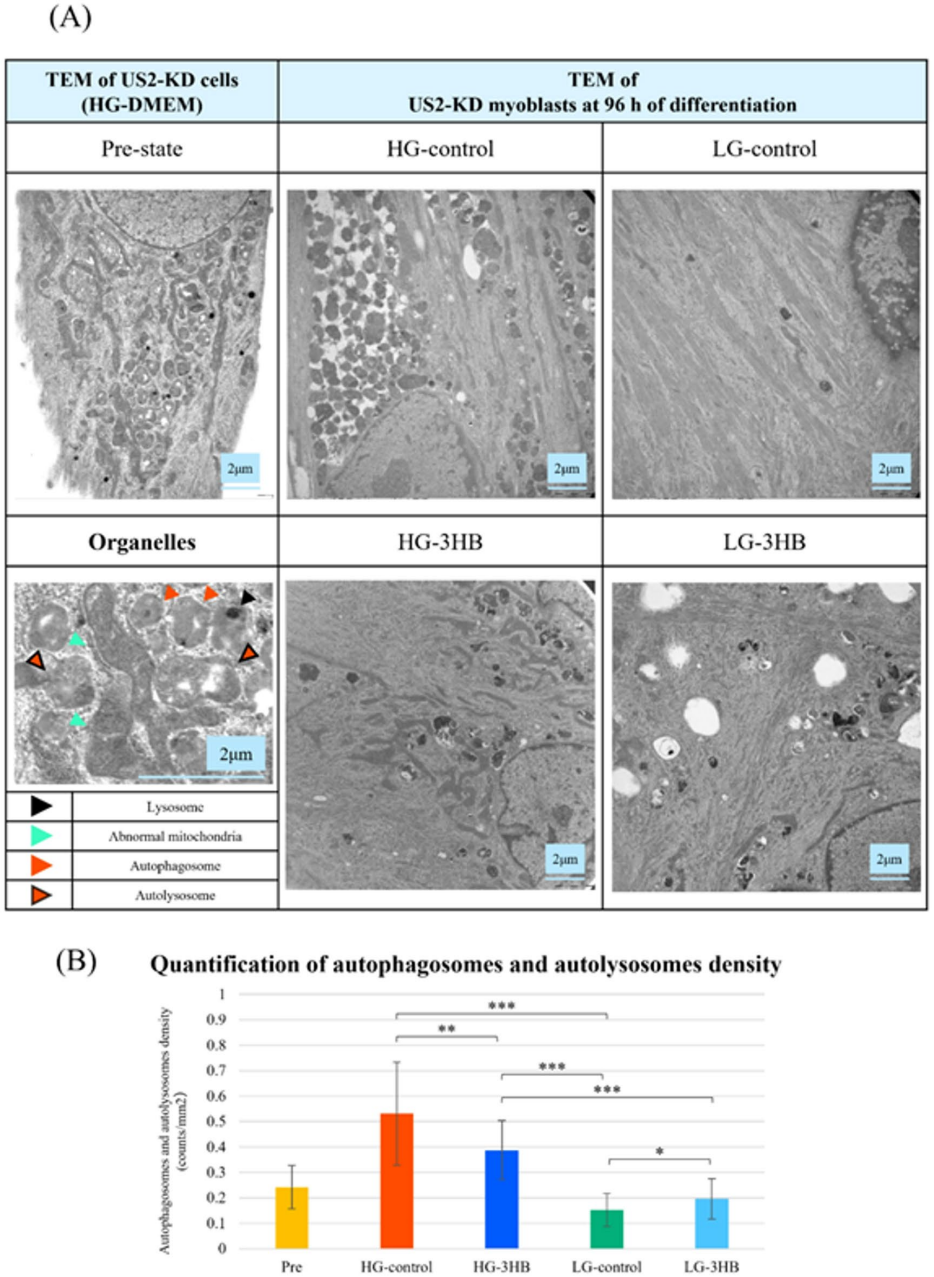
Transmission electron microscopy reveals medium and 3HB-dependent autophagosomes and autolysosomes accumulation during US2-KD myoblast differentiation. (**A**) TEM images of US2-KD cells cultured in high-glucose DMEM at the pre-differentiation state (Pre) and at 96 h of differentiation under high-glucose control (HG-control) and low-glucose control (LG-control) conditions, as well as representative organelle morphology under HG-3HB and LG-3HB conditions. Lysosomes (black arrowheads), abnormal mitochondria (cyan arrowheads), autophagosomes (orange arrowheads), and autolysosomes (red arrowheads) are indicated. Scale bars: 2 μm (original magnification ×10,000). (**B**) Quantification of autophagosomes and autolysosome density (counts/µm²) in US2-KD cells under each condition (Pre, HG-control, HG-3HB, LG-control, and LG-3HB). Data are presented as mean ± SD (*n* = 3). *p < 0.05, **p < 0.01, ***p < 0.001. Representative annotations of these autophagy-related organelles are also shown at higher magnification in Supplementary Figure S3. TEM, transmission electron microscopy; DMEM, Dulbecco’s modified Eagle’s medium; 3HB, 3-hydroxybutyrate; SD, standard deviation; HG, high-glucose; LG, low-glucose.

Figure 5A shows TEM images of US2-KD myotubes in the Pre state and after 96 h of differentiation. In the Pre state, numerous autophagosomes-autolysosomes and abnormal mitochondria were observed in the cytoplasm of the cells. This feature was not specific to US2-KD cells, as a similar ultrastructural pattern was also observed in the female rectus abdominis myoblast line HU5-KD (Supplementary Fig. 2)^18^. Under LG conditions, autophagosomes-autolysosomes were markedly decreased compared to those under HG conditions, and mitochondrial morphology appeared more normal. In addition, myofibril-like ultrastructures were substantially thicker than those in the HG group, indicating maturation of the contractile apparatus. Regarding the effects of 3HB, in the HG groups, 3HB treatment was associated with a reduction in autophagosomes-autolysosomes, whereas in the LG group, it resulted in greater persistence. Figure 5B shows quantitative data for autophagosome–autolysosome density. This density was lower in LG-control than in HG-control (*p* < 0.001), reduced in HG-3HB versus HG-control (*p* < 0.01), and higher in LG-3HB than in LG-control (*p* < 0.05).

**Fig. 6:**
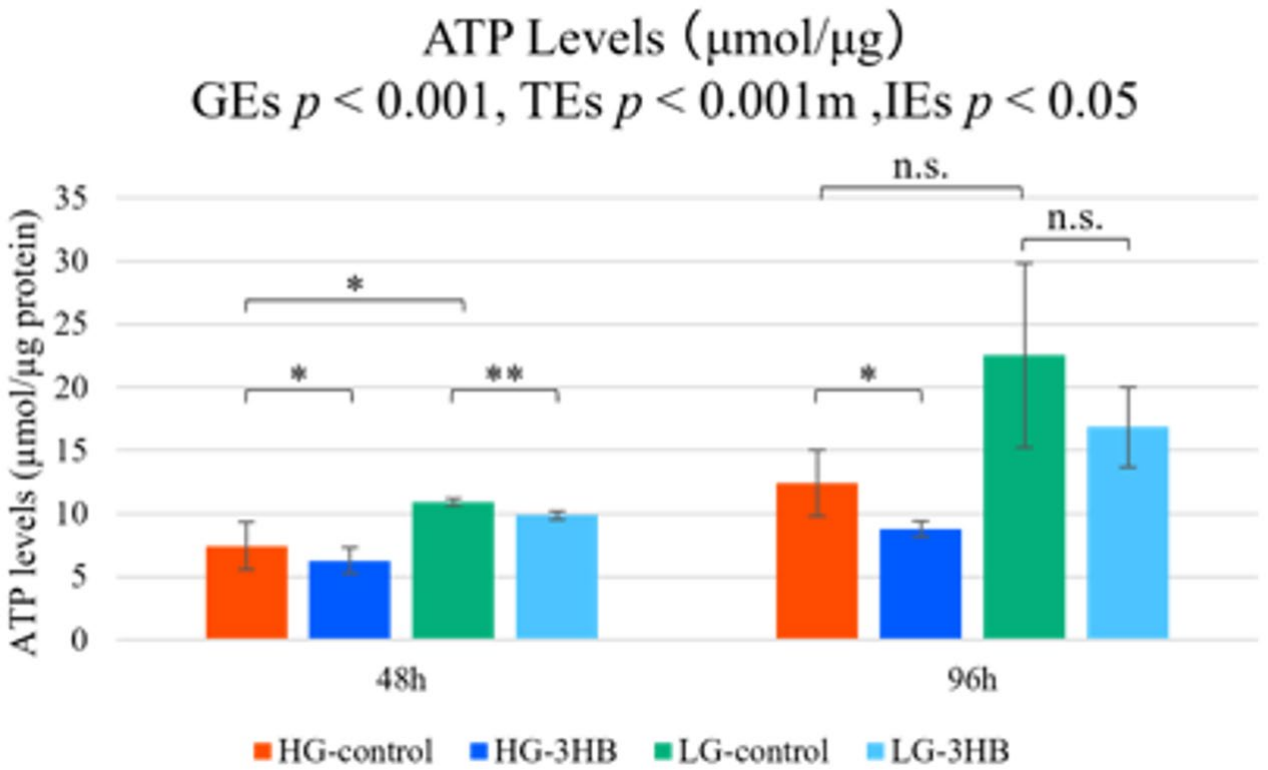
Intracellular ATP levels during US2-KD myoblast differentiation under different medium conditions. Intracellular ATP levels in US2-KD cells cultured under high-glucose control (HG-control), high-glucose plus 3-hydroxybutyrate (HG-3HB), low-glucose control (LG-control), and low-glucose plus 3-hydroxybutyrate (LG-3HB) conditions at 48 h and 96 h after induction of differentiation. ATP concentration (µmol/µg protein) was measured using a luminescence-based ATP assay and normalized to total protein content. Data are presented as mean ± SD (*n* = 3). *p < 0.05, **p < 0.01; n.s., not significant (two-way ANOVA with post hoc tests for group and time effects). 3HB, 3-hydroxybutyrate; SD, standard deviation; HG, high-glucose; LG, low-glucose; ANOVA, analysis of variance.

The ATP levels are shown in Fig. 6. Two-way ANOVA revealed significant main effects of glucose concentration (GEs, *p* < 0.001) and time (TE, *p* < 0.001). At 48 h, ATP content was significantly higher in LG-control than in HG-control, whereas at 96 h it tended to be higher in LG-control, but the difference was not statistically significant. In comparisons between HG-control and HG-3HB, ATP levels were significantly higher in HG-control at both 48 and 96 h. Between LG-control and LG-3HB, ATP levels were significantly higher in LG-control at 48 h, while at 96 h they showed a similar trend without reaching statistical significance. Analysis using MT-1, a fluorescent probe that reflects abnormalities in the mitochondrial membrane potential, showed a tendency toward lower values in the LG group than in the HG group; however, this difference did not reach statistical significance. Intracellular ROS levels also did not differ significantly among the groups, but tended to be lowest in LG-control, followed by LG-3HB, HG-3HB, and HG-control (Supplementary Fig. S4).

Finally, the results of the MYHC immunostaining are shown (Fig 0.7). MYHC-positive myotubes were observed in all groups, but the staining intensity differed depending on the culture conditions. Quantitative analysis of the mean gray value revealed that MYHC staining intensity was highest in LG-control, followed by LG-3HB, HG-3HB, and HG-control.

## Discussion

In this study, we compared a high-glucose DMEM (nutrient-enriched medium, NEM) with a low-glucose MEM (physiological medium, PM) to assess how the metabolic milieu and 3HB influence hEUS myogenic differentiation. We found that simply switching to the PM condition had a much stronger impact on differentiation than adding 3HB itself.

### Effects of glucose concentration and 3HB on myogenic differentiation

Phase-contrast microscopy (Fig. 1) revealed that under HG conditions, myotube formation in US2-KD cells progressed slowly, with well-developed myotube structures evident at 144 h. This differentiation time course was broadly consistent with previous reports that US2-KD cells reached a differentiation peak at 144 h and subsequently underwent myotube detachment at approximately 196 h in HG medium^13^. In contrast, HG-3HB cells did not display any obvious differences from HG-control cells in the phase-contrast images. Under LG conditions, however, differentiation was accelerated, and extensive myotube formation was observed at 96 h. At 144 h, most myotubes had disappeared in LG-3HB, whereas in LG-control, myotubes were reformed from residual myoblasts following detachment, yielding a morphology reminiscent of secondary differentiation. These findings suggest that, in hEUS myoblasts, adjustment to PM promotes earlier and more dynamic myogenic remodeling more effectively than ketone body supplementation or exposure to NEM per se.

Even though 3HB produced only modest morphological changes, it raised important questions about whether it mainly functions as an energy source or as a signaling metabolite^15–17^, and why the culture medium so strongly shapes the differentiation phenotype. To explore this background, we performed nontargeted metabolomics on culture supernatants from all four groups. Statistical analyses showed that extracellular metabolic profiles were separated predominantly by medium rather than by 3HB, indicating that medium composition is the primary determinant, with 3HB acting as a context-dependent modifier on this background (Fig. 2).

US2-KD cells underwent dynamic metabolic remodeling during differentiation, with the dominance of individual energy and amino acid–related pathways shifting over time, indicating that myogenesis proceeds through stepwise changes in pathway usage^19^. The relative contribution of these pathways was clearly segregated by medium type and 3HB exposure, implying that distinct metabolic states arise within an ostensibly similar differentiation process. In particular, differentiation in HG cultures advanced in a largely unidirectional manner up to 144 h, whereas the 96–144 h interval in LG cultures appeared to represent a second differentiation phase, accompanied by transient reorganization of signaling (Supplementary Tables S1-S4).​.

In LG-control cells, pathways associated with mitochondrial function and intracellular quality control, such as branched-chain amino acid metabolism, one-carbon metabolism, and pantothenate/CoA biosynthesis, were preferentially enriched in the early phase, consistent with a “quality control–prioritized differentiation mode” in which autophagy and mitochondrial quality control are established early and maintained throughout differentiation^20–22^.

By contrast, HG-control cells showed prominent enrichment of taurine/hypotaurine, cysteine/methionine, and glutathione metabolism, together with lipid- and membrane-related pathways, suggesting that under NEM conditions a baseline state dominated by oxidative stress defense and membrane remodeling is established^21–23^.​.

With 3HB supplementation, HG cultures exhibited further enrichment of redox- and stress-related pathways, including cysteine/methionine metabolism, glutathione metabolism, and arginine biosynthesis, whereas LG cultures preferentially engaged amino acid, nucleotide, and phospholipid signaling pathways, such as arginine/proline metabolism, inositol phosphate metabolism, purine and pyrimidine metabolism, and vitamin B6–related metabolism. Collectively, these patterns suggest that 3HB acts as a context-dependent modulator that tunes intracellular networks into states that support differentiation control alongside autophagy and mitochondrial quality control^15–17^.

This notion agrees with reports that glucose restriction in C2C12 myotubes activates autophagy and metabolic remodeling, highlighting tight coupling between nutrient status and quality control in skeletal muscle cell^24^.

In a previous study using hEUS myoblasts, we performed nontargeted metabolomics and demonstrated that the TCA cycle functions as a central metabolic hub during the differentiation of these cells^13^. Building on this finding, the present study also focused on the TCA cycle as the core framework for analyzing energy metabolism (Fig. 3). Because ketone bodies are generally utilized as alternative energy substrates during fasting, and this property underlies the rationale for ketogenic diets, we initially hypothesized that the predominant effects of 3HB would be exerted at the level of energy metabolism, predicting that the TCA cycle would be more strongly activated under LG than under HG conditions^25,26^. However, the present results diverged substantially from this hypothesis. Neither the HG nor the LG cultures exhibited a consistent temporal pattern in TCA cycle intermediates. In HG cultures, only isocitrate and malate showed significant group effects, with many intermediates tending to increase upon 3HB supplementation, whereas in LG cultures, a significant group effect was observed only for α-ketoglutarate, and most other intermediates tended to decrease. These findings indicate that under the HG and LG conditions employed in this study, even high concentrations of 3HB do not primarily promote energy production via unidirectional enhancement of TCA cycle activity. Instead, 3HB appears to act predominantly as a context-dependent modulator of the signaling pathways and quality control–related metabolic networks^15–17,20,27–30^.

As summarized in Table 1, both HG and LG cultures showed significant changes, with 3HB exposure and time, in multiple metabolites related to autophagy, mitochondrial function, and epigenetic regulation^20,22,25,28,31–33^. In contrast, alterations in classical energy-related metabolites were relatively limited, whereas a comparatively large number of metabolites at the signaling and quality control levels were affected^19,23,29,34^. Overall, HG conditions were characterized by changes in metabolites associated with mitochondrial function and oxidative stress responses, along with notable fluctuations in monoaminergic signaling molecules, such as catechol, dopamine, and serotonin. By comparison, characteristic changes were observed in oleamide, which has been linked to muscle autophagy and antiatrophy effects, and p-octopamine, which is associated with nutrient and stress signaling, suggesting that 3HB preferentially modulates networks related to either mitochondrial homeostasis or autophagy/quality control, depending on the culture conditions^29,30,35,36^. Although limited in number, aromatic amino acid–derived organic acids, such as phenylpyruvic acid and phenyllactic acid, showed significant group effects across both the HG and LG cultures^19,23,34^. This finding suggests that 3HB exposure may subtly influence the transcriptional environment via phenylalanine/tyrosine metabolism and redox balance, thereby gradually modulating the expression programs of myogenic genes, such as *MYOG* and *MYH7*, in line with the gene expression results presented below^15,17,19,25,31–33,37^.

Gene expression analysis revealed that the myogenic markers *MYOG* and *MYH7* were induced earlier and more strongly in LG cultures than in HG-control, indicating that LG medium favors both the acceleration of morphological differentiation and the earlier activation of differentiation programs^13^. The effects of 3HB on gene expression were modest under HG conditions: 3HB tended to slightly enhance late *MYH7* expression, but this did not reach statistical significance, suggesting that in NEM conditions, 3HB may only mildly support late-stage maturation^15,25^. Under LG conditions, however, 3HB supplementation resulted in significantly higher *MYH7* expression at 48 h compared with LG-control, and although absolute expression levels were low, *DES* also tended to be higher in LG-3HB than in LG-control (Fig. 4). *MYOG* expression was slightly higher in LG-3HB than in LG-control at 48 h, albeit not significantly, and became significantly higher in LG-3HB at 96 h (Fig. 4). At 144 h, *MYH7* expression in LG-3HB was slightly lower than in LG-control, and together with the phenotypic and metabolomic findings, these data suggest that, although 3HB does influence *MYOG* expression to some extent, factors other than *MYOG* are also involved in determining the final differentiation phenotype (Figs. 1, 2, 3 and 4) ^36–38,42^.

The expression of the autophagy-related gene LC3B closely paralleled changes in MYH7 when the HG and LG groups were compared^20^^23,27–30,38^. At 48 and 96 h, LC3B expression was higher in the LG group than in HG-control, reaching statistical significance at 48 h and showing a similar trend at 96 h, whereas at 144 h it tended to be higher in the HG group than in the LG group. With respect to the effects of 3HB, both HG-3HB and LG-3HB tended to show higher LC3B expression than their respective control groups at 48 h, while at 96 h LG-control exhibited the highest levels.​.

Taken together, these findings indicate that, in this batch, culture medium exerted a strong influence: at the level of extracellular metabolomics, HG and LG showed marked differences in metabolic profiles, with substantial changes in pathways including the TCA cycle and in signaling-related metabolites depending on the presence or absence of 3HB, whereas qRT-PCR detected only limited 3HB-dependent changes other than LC3B^14,18,19,23,34^. These observations suggest that medium composition has a major impact that is directly reflected in the differentiation phenotype, while the effects of 3HB are more restricted and may act mainly through autophagy-related signaling^15,16,20,24,27–30^. However, because current autophagy guidelines regard LC3B mRNA expression only as a supportive indicator, an additional differentiation batch was examined by TEM to obtain morphological confirmation of autophagic activity^38^.

### Autophagic activity and organelle remodeling during differentiation

Organelle remodeling during skeletal muscle differentiation was further clarified using TEM, which revealed a strong dependence on the culture conditions^12,13,20,24,27–30,38^. In myotubes differentiated under HG-control conditions, myofibril-like ultrastructures remained poorly aligned and thin, and numerous autophagosomes-autolysosomes density was significantly lower in LG-control than in HG-control (*p* < 0.001), and morphologically abnormal mitochondria were observed in the cytoplasm, suggesting persistent cellular stress and delayed cytoplasmic remodeling^20,23,24,27–30^, . In contrast, myotubes in LG-control displayed well-developed, regularly aligned myofibril-like ultrastructures, few autophagy-related structures, and more uniform mitochondrial morphology, indicating that remodeling toward a mature contractile apparatus proceeds efficiently under physiological culture conditions^20,27–29^.

The impact of 3HB also differed markedly depending on the culture conditions^14–17,25^. Under HG conditions, 3HB treatment reduced the number of autophagosomes-autolysosomes compared to HG-control, suggesting that 3HB can alleviate organelle-level stress responses and the abnormal accumulation of autophagy-related structures^15–17,20,24,27–30^. In contrast, under LG conditions, the overall myofibril-like ultrastructures was largely preserved in the presence of 3HB, but the alignment of myofibril-like ultrastructures was less organized, and autophagosomes-autolysosomes were relatively more frequent than in LG-control. Thus, although LG-3HB showed a marked reduction in autophagosomes-autolysosomes compared with HG-control, it appeared to interfere with the shift toward more efficient progression of autophagy and optimal alignment of myofibril-like ultrastructures compared with the more physiological LG-control condition^20,24,27–29,38^.

These ultrastructural findings support the notion that, relative to NEM, PM facilitates more efficient progression of autophagy and promotes both myofibril-like maturation and organelle remodeling, consistent with the *LC3B* expression changes detected by qRT-PCR^20,24,27–29,38^. Although the phenotypic eff ects of 3HB were not always evident inthe batches used for Figs. 1–4 and Table 1, its infl uence was more clearly captured in an independent TEM batch,suggesting that 3HB may act as a culture condition–dependent regulator that can, in some contexts, contribute tothe correction of autophagy and mitochondrial quality control, while in others, it may hinder their optimal tuning.^14–17,20,24,27–29^.

Intracellular ATP levels were significantly higher under PM conditions than under NEM conditions at 48 h, and tended to remain higher at 96 h, suggesting that these ultrastructural changes are accompanied by improved mitochondrial quality and energy production (Fig. 6, Supplementary Fig.S4)^12,13,27,29,34^.

**Fig. 7:**
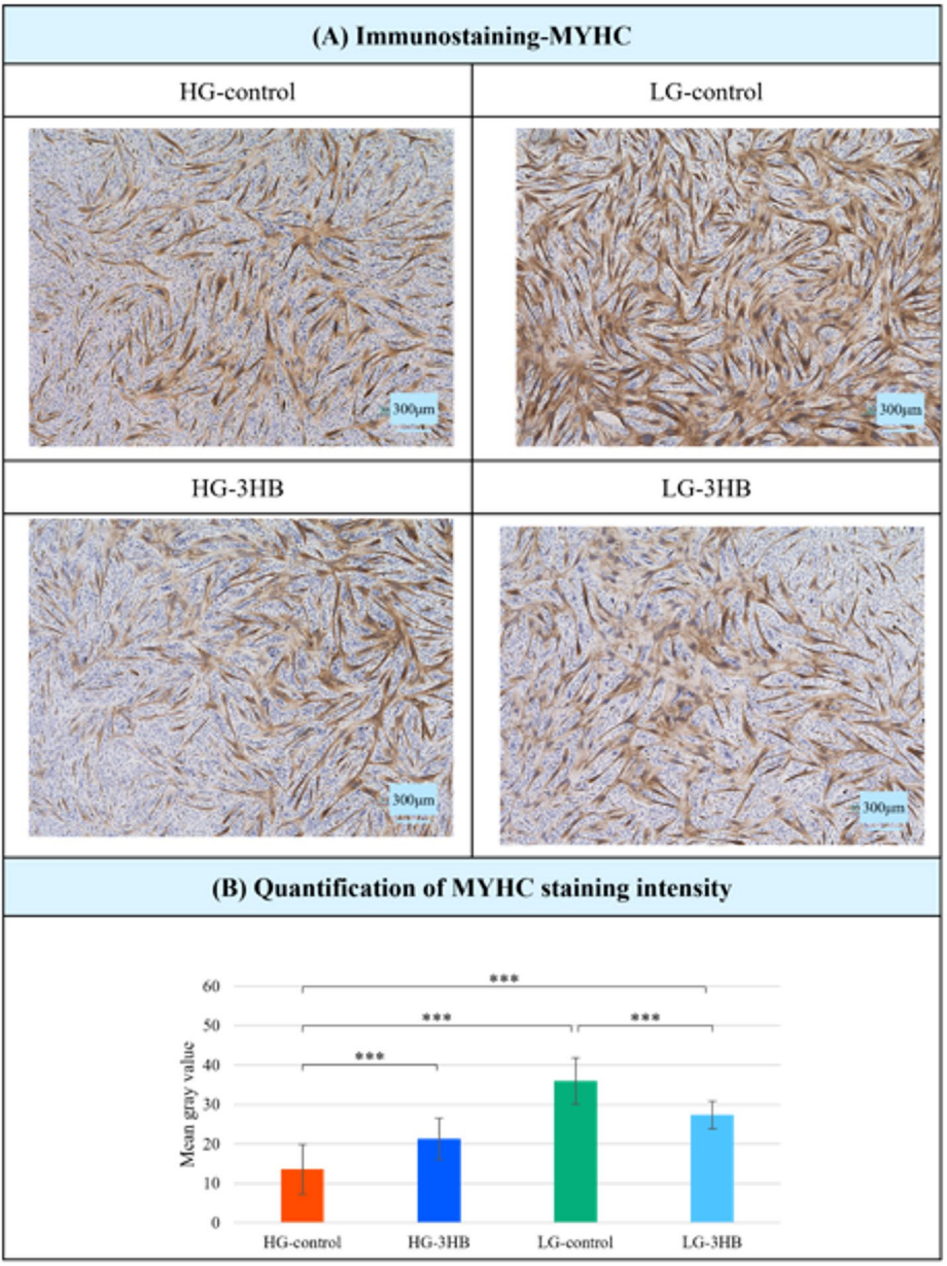
Immunostaining and quantification of MYHC under different culture conditions. (**A**) Representative immunostaining of MYHC in US2-KD cells cultured under high-glucose control (HG-control), high-glucose with 3-hydroxybutyrate (HG-3HB), low-glucose control (LG-control), and low-glucose with 3-hydroxybutyrate (LG-3HB) conditions. Images were acquired 144 h after induction of differentiation. Scale bar: 300 μm (original magnification ×40). (**B**) The bar graph shows the mean gray value of MYHC staining intensity quantified for each condition, and data are presented as mean ± SD (*n* = 3). ***p < 0.001. a.u., arbitrary units; 3HB, 3-hydroxybutyrate; SD, standard deviation; HG, high-glucose; LG, low-glucose.

Immunostaining for MYHC (Fig. 7) showed that the expression of this differentiation marker was highest in LG-control, followed by the LG-3HB, HG-3HB, and HG-control, a pattern that was broadly consistent with the TEM findings and autophagosomes-autolysosomes quantification^19,31–33,37^. These results indicate that the formation of the contractile apparatus was most advanced under PM conditions, and they also revealed differences between HG-control and HG-3HB that were not clearly discernible by phase-contrast microscopy alone (Fig. 1)^13,19,31–33,37^.

### Effects of different media and 3HB on myotube maturation

These findings indicate that PM-based conditions are more suitable than NEM-based conditions for the differentiation of external urethral sphincter muscle cells^12,13,18,19,37^. A likely explanation is that PM better supports both metabolic and quality control–type autophagy, thereby enhancing overall autophagic activity^20,24,27–30,38^. Under NEM conditions, 3HB clearly increased MYHC expression and was associated with some improvement at the organelle level, but these changes did not translate into obvious gains in myotube morphology^14–17,20,24,28–31^. By contrast, under PM conditions, PM alone improved organelle integrity and yielded the most advanced myofibril-like ultrastructures whereas 3HB produced only modest additional effects and in some respects seemed to disturb optimal myofibril-like alignment and autophagy-related structures^12,13,20,24,27–30^. Taken together, this pattern suggests that, under the PM conditions used here, energy metabolism, autophagy, and mitochondrial quality control are already tuned close to an optimal balance, so extra 3HB acts more as a mild perturbation than as a strong promoter of differentiation^14–17,20,24–30^.

Nevertheless, even under such PM conditions, it remains possible that other metabolic or signaling interventions, distinct from 3HB, could further promote myofibril-like maturation and organelle quality control.

### Clinical implications of PM and 3HB for hEUS myogenesis

NEM is widely used for cell culture but resembles the nutrient-rich environment seen in obesity and diabetes, whereas PM is thought to more closely reflect the physiological milieu of healthy individuals ^20–22,26−28,30,39,44^. In this study, PM was associated with a more appropriately maintained autophagic activity, better preservation of mitochondrial function and removal of damaged organelles, and higher MYHC expression, whereas NEM alone did not fully correct these abnormalities^12,13,19,20,24,27–30,38^. Maintaining a PM-like metabolic and autophagic state therefore appears most favorable for myogenic differentiation and muscle function, and in everyday life relies mainly on regular exercise and a balanced diet, although long-term adherence is challenging^22,23,25,28,31,32,37^.​ Clinical studies indicate that 3HB can help reduce muscle atrophy and promote weight loss in patients with metabolic disorders, but in healthy subjects it does not consistently enhance muscle strength and may even impair muscle function in some settings^14–17^^25,26,39^. Taken together, these observations suggest that 3HB provides limited additional benefit when a PM-like, metabolically favorable state is already maintained, but under NEM-like metabolic stress it may serve as a means to support organelle quality control and muscle function^14–17,20,25,27–30,40,41^. Our findings further suggest that preserving a PM-like metabolic and organelle quality control state is particularly important in stress urinary incontinence, which is common in people with diabetes or obesity, and that 3HB may partially support this state^1–5,7–9,39,40,42,43^.

### Limitations

This study has several limitations. First, we examined how culture conditions and metabolic remodeling affect hEUS myoblast differentiation, but we could not directly assess the final caliber of myotubes or muscle fibers. The immortalized cell line was derived from the external urethral sphincter of a single male donor, which restricts the generalizability of the findings across different sexes, ages, and clinical backgrounds. Thus, our culture model cannot fully reproduce the complex in vivo microenvironment, and extrapolation to hEUS biology in vivo should be made with caution^3,37,44,45^.​ We also performed GC–MS–based metabolomic analysis of culture supernatants to capture overall changes in extracellular metabolic flux, but this approach does not directly represent intracellular metabolism^13,18,19^. Integrating these measurements with real-time bioenergetic assays, such as mitochondrial respiration and glycolytic activity, would strengthen the interpretation of TCA cycle–related changes^29,34,46^. The metabolomic platform used here also has inherent limits in sensitivity and metabolite coverage, and may not have detected all relevant metabolites and pathways^13,18,19^.​ Finally, although TEM clearly visualized autophagosomes-autolysosomes and the remodeling of mitochondria and myofibril- ultrastructures, we did not fully clarify how these morphological changes are mechanistically linked to upstream signaling pathways via downstream effectors such as LC3-I/II, FOXO, mTORC1, AMPK, and early myogenic regulators including MyoD. Future studies should integrate these signaling nodes with the autophagy machinery to define the regulatory network from the metabolic environment, through signal transduction, to autophagy-driven organelle remodeling^20,24,27–30,38,47^.

## Conclusion

In this study, the differentiation behavior of immortalized human external urethral sphincter (hEUS) myoblasts (US2-KD) was strongly dependent on the culture conditions, with low-glucose MEM exerting a more pronounced pro-differentiation effect than 3-hydroxybutyrate (3HB). Differentiation and ultrastructural maturation, including autophagy-linked remodeling of mitochondria and myofibril-like ultrastructures were more effectively promoted by shifting the culture conditions toward a more physiological state than by simply adding 3HB under nutrient-enriched conditions. These findings suggest that recreating a healthy metabolic environment with appropriate autophagic activity is a primary determinant of hEUS myogenic and organelle remodeling, whereas 3HB functions not as a standalone driver but as a context-dependent metabolic modulator.

## Methods

### Ethics information

All procedures involving human participants were conducted in accordance with the ethical standards of the institutional and/or national research committees and with the 1964 Declaration of Helsinki and its later amendments or comparable ethical standards. The study protocol was approved by the Institutional Review Board of Oita University (approval number: 307). Written informed consent was obtained from all donors who provided tissue samples for establishment of the cell line. The US2-KD cell line used in this study was derived from a single male donor. No new participants were recruited for this study. All experiments were conducted in vitro using a previously established immortalized human myoblast cell line. No animals were used, and no animal experiments were performed in this study.

### Cell culture

The male human external urethral sphincter cell line (US2-KD) used in this study was generated as described previously. Primary myogenic cells were isolated from external urethral sphincter tissue and immortalized by introducing genes encoding a mutant form of cyclin-dependent kinase 4, cyclin D1, and human telomerase reverse transcriptase (TERT)^13,43^. This procedure allowed long-term propagation of cells while preserving their myogenic differentiation capacity. US2-KD cells were seeded in type I collagen-coated six-well plates (Corning Life Sciences, Corning, NY, USA) at a density of 4 × 10⁵ cells per well. Cells were cultured in a growth medium (GM) composed of HG-DMEM (4.5 g/L) (Thermo Fisher Scientific, Waltham, MA, USA) supplemented with 20% fetal bovine serum (FBS; Sigma-Aldrich, St. Louis, MO, USA) and 2% Ultroser G serum substitute (Cat. no. 15950-017, Cytiva, Marlborough, MA, USA) for the first 24 h. HG-DMEM is widely used as a standard basal medium for mammalian cell lines and was therefore employed as the reference control condition in this study^48–50^. This time point, termed the prestate, marked the end of the initial growth phase before the induction of differentiation, at which point GM was replaced with differentiation medium (DM). For myogenic differentiation, US2-KD cells were cultured under four conditions: (1) high-glucose DMEM (4.5 g/L; HG-control), (2) high-glucose DMEM with 10 mM 3HB (HG-3HB), (3) low-glucose MEM (Thermo Fisher Scientific,1 g/L; LG-control), and (4) low-glucose MEM with 10 mM 3HB (LG-3HB). The differentiation medium for each condition contained 2% FBS, 5 µg/mL holo-transferrin, 0.5 µg/mL insulin, and 10 nmol/L sodium selenite (all from Sigma-Aldrich, St. Louis, MO, USA). The cultures were maintained at 37 °C in a humidified atmosphere of 5% CO₂. The differentiation medium was replaced 48 and 96 h after seeding (Supplementary Fig. S5A). To determine the optimal 3HB concentration, preliminary differentiation assays were performed in HG-DMEM using 5 and 10 mM 3HB, which are concentrations commonly used in previous in vitro studies. *MYH7* expression at 144 h was higher with 10 mM than with 5 mM 3HB (Supplementary Fig. S6A). Cytotoxicity testing from 5 to 10 mM showed no significant toxicity up to 10 mM, based on the cell counts at 48 h (Supplementary Fig. S6B). Therefore, 10 mM 3HB was used in the subsequent experiments. Culture supernatants and cells were collected every 48 h after differentiation induction (48, 96, and 144 h). This 48-hour interval was selected because preliminary experiments showed clear morphological changes and robust induction of the myogenic marker *MYOG* at these time points during myoblast differentiation, whereas more frequent sampling (every 24 h) caused mechanical and metabolic stress that reduced cell viability and reproducibility. In contrast, longer intervals risk missing important temporal changes during differentiation of the cells. Thus, a 48-hour sampling interval provided a suitable balance between capturing the dynamics of differentiation and maintaining cell health and experimental consistency. To clarify the nutritional background of the culture media used in this study, it should be noted that DMEM contains higher concentrations of amino acids and vitamins than MEM, and that the high-glucose formulation (4.5 g/L) greatly exceeds the physiological glucose levels. HG-DMEM is hereafter referred to as nutrient-enriched medium (NEM), representing a high-nutrient condition. In contrast, LG-MEM is hereafter referred to as the physiological medium (PM) and was adopted, in a relative and convenient sense, as a comparator medium that reflects the state of non-over-nourished healthy adults because its sugar and amino acid/vitamin compositions more closely resemble in vivo conditions. Our use of the term “physiological” was not intended to imply an absolute reproduction of in vivo conditions, but rather to indicate a relative position in comparison with high-glucose DMEM^51^. The compositions of DMEM and MEM are shown in Supplementary Tables S5 and S6.

For each condition, phase-contrast imaging, metabolomic sampling of culture supernatants, and collection of cells for RNA extraction were performed in parallel from the same cultures at Pre, 48, 96, and 144 h.

Phase-contrast imaging, metabolomic profiling of culture supernatants, and Quantitative Real-time (qRT)-PCR were performed on samples collected in parallel from the same batch of US2-KD cultures at 48, 96, and 144 h under each condition. In contrast, Transmission electron microscopy (TEM), ATP measurements, mitochondrial ROS assays, and immunostaining were performed in separate independent cultures of US2-KD cells under the same four conditions, with samples collected at time points optimized for each assay.”

### Gas chromatography–mass spectrometry (GC–MS)

The metabolites in the culture supernatant were profiled using a GCMS-TQ8030 system (Shimadzu Corporation, Kyoto, Japan). The culture supernatants were subjected to methanol–water–chloroform extraction, and 2-isopropylmalic acid was added as an internal standard. Chromatographic separation was achieved using a DB-5 capillary column under conventional GC–MS conditions, and individual metabolites were annotated using the SHIMADZU Smart Metabolite Database. Peak detection and quantification were performed using GCMS solution version 4.53 (Shimadzu Corporation), and the relative peak area of each metabolite was used as a surrogate index of its abundance. At each time point, supernatants were collected in triplicate (*n* = 3) from independently cultured samples for further analysis. For the 0 h (Pre) time point, supernatants were harvested immediately after replacing GM with DM; thus, pre-samples represented DM-only controls without prior incubation period. At each sampling point, the culture supernatants were collected and centrifuged at 1,600 rpm for 5 min at 4 °C to remove detached cells and debris. The clarified supernatants were transferred to fresh tubes and stored at − 80 °C until analysis (Supplementary Fig. S5B).

The numbers in the Venn diagram represent metabolites that remained after pathway annotation and statistical filtering and are therefore smaller than the number of initially quantified peaks because unannotated or redundant signals were removed from the data set.

The raw GC–MS peak area data for each metabolite are presented in Supplementary Tables S7 and S8.

### Metabolomics data preprocessing and normalization

Metabolomics data were normalized using two different procedures depending on the analytical purpose. First, for multifactorial metabolomic analyses involving culture medium conditions and the presence or absence of 3HB, a glass-type delta (Δ) was calculated for each metabolite using the Pre group as a reference. Specifically, the relative intensity at each time point was centered by subtracting the mean relative intensity of the Pre group and then divided by the standard deviation of the Pre group, thereby expressing the change as the number of standard deviations relative to the variability in the Pre group. In contrast, because the baseline differences between the medium conditions were substantial, pathway analyses to evaluate the effects of 3HB and pairwise comparisons of individual metabolites were performed after Z-score transformation (mean 0, standard deviation 1) within the HG and LG groups, respectively, to harmonize the scales of the data. This two-step normalization strategy enabled (1) an effect size–like evaluation of temporal changes relative to the Pre group and (2) a consistent comparison of 3HB effects within each medium condition^52,53^.

### Quantitative real-time (qRT)-PCR

After collecting the culture supernatants for metabolomic analysis, the cells were harvested in biological triplicates for RNA extraction. Total RNA was analyzed by quantitative real-time PCR using a LightCycler 96 version 1.1.0.1320 (Roche Diagnostics, Indianapolis, IN, USA) to assess *MYOG* (Cat. no. QT00001722), *MYH7* (Cat. no. QT00000602), *DES* (Cat. no. QT00071778), *LC3B* (Cat. no. QT01673007), and *GAPDH* (Cat. no. QT01192646). Relative gene expression was calculated using the ΔΔCt method with *GAPDH* as the internal reference. All primers were obtained from QIAGEN (Hilden, Germany).

### Transmission electron microscopy (TEM)

For TEM analysis, US2-KD cells were cultured and differentiated under the four conditions described above. Pre state cells (0 h) and differentiated cells at 96 h were fixed and processed for TEM, and three independent samples were prepared for each condition (*n* = 3). Cells were fixed in 2.5% glutaraldehyde in buffer for 1 h at room temperature and post-fixed with 1% osmium tetroxide. After en bloc staining with 4% uranyl acetate, the samples were dehydrated using a graded series of ethanol and propylene oxide and embedded in an epoxy resin. Ultrathin sections (~ 60–80 nm) were cut using a diamond knife, mounted on copper grids, and stained with 4% uranyl acetate and lead citrate. To minimize observer bias, image acquisition was performed by an independent researcher who was blinded to the experimental groups. For each sample, ten non-overlapping fields were randomly selected and imaged at 10,000× magnification. The density of autophagosomes and autolysosomes were manually counted in the TEM images using ImageJ (version 1.54 g, NIH, USA) and normalized to the cytoplasmic area (counts/µm²), with autophagosomes identified as double-membrane structures containing relatively intact cytoplasmic material or organelles, and autolysosomes defined as autophagosomes that have fused with lysosomes and contain partially degraded cytoplasmic components and organelles within a single limiting membrane.

### Intracellular ATP measurement

Because 3-hydroxybutyrate (3HB) can act both as an alternative energy substrate and as a signaling metabolite in skeletal muscle cells, intracellular ATP content was measured as a global indicator of cellular energy metabolism and mitochondrial function during differentiation under each culture condition^14–17^.

Intracellular ATP levels in US2-KD cells were quantified using an ATP Assay Kit-Luminescence (Dojindo Molecular Technologies, Kumamoto, Japan). After removing the culture medium and washing once with phosphate-buffered saline to eliminate residual medium and extracellular metabolites, the cells were collected in serum-free DMEM or MEM and homogenized to extract intracellular components. Luminescence was measured using a microplate reader, and ATP concentrations were determined from a calibration curve generated using ATP standards supplied with the kit. ATP levels were assessed at 48, 96 h after the induction of differentiation, and measurements were performed in triplicate for each culture condition. ATP concentrations were normalized to the total protein content of each sample, which was determined using the Bradford assay (Cat. no. 23200, Thermo Fisher Scientific).

### Mitochondrial reactive oxygen species (ROS) measurement

Mitochondrial superoxide levels were detected using MitoBright ROS Deep Red (MT-1,Dojindo Laboratories, Kumamoto, Japan). The dye was stored at 0–5 °C. A 10 mM stock solution was prepared by dissolving 100 nmol of MitoBright ROS Deep Red in 10 µL of DMSO and stored at − 20 °C for up to one month. Before use, the stock solution was diluted 1,000-fold with culture medium or Hank’s Balanced Salt Solution (HBSS) to prepare a 10 µM working solution. Cells were seeded into the appropriate culture vessels and incubated overnight at 37 °C under 5% CO2. The culture medium was removed, and the cells were washed once with fresh medium or a HBSS balanced salt solution. The cells were then incubated with MitoBright ROS Deep Red working solution at 37 °C and 5% CO2 for 30 min. Following incubation, the working solution was removed, and the cells were washed twice with HBSS balanced salt solution. Fluorescence was measured immediately using fluorescence microscopy, a plate reader, and flow cytometry FACSCalibur; BD Biosciences (San Jose, CA). The excitation/emission settings were adjusted according to the detection instrument: excitation at 535–565 nm and emission at 660–690 nm for plate readers; excitation at 561–633 nm and emission at 640–700 nm for microscopy; and excitation at 633 nm and emission at 640–700 nm for fluorescence-activated cell sorting. For multiplex staining involving red fluorophores, 633 nm excitation was used to minimize spectral overlap. For plate-reader measurements, cells were seeded in 96-well plates.

### Immunostaining

Cells were fixed in 100% methanol for 20 min at room temperature and permeabilized. After rinsing with PBS, nonspecific binding was blocked by incubating the slides with 10% normal goat serum (Cat. no. 06-349-64, Nacalai Tesque, Inc., Kyoto, Japan) in PBS for 30 min. The specimens were subsequently incubated overnight at 4 °C with an anti-MYHC primary antibody (Cat. no. 05-716-I-25UL, MilliporeSigma). The following day, the slides were treated with EnVision anti-mouse secondary antibody (Cat. no. K4001, Agilent Technologies, Dako Denmark A/S, Glostrup, Denmark) for 30 min at room temperature. Signal development was performed for 10 min using the DAB substrate (Cat. no. K3468, Agilent Technologies, Dako Denmark A/S), and the nuclei were counterstained with hematoxylin (Cat. no. 30002; Muto Pure Chemical Industries, Ltd., Tokyo, Japan) and coverslipped with CC/Mount (Cat. no. K002, Diagnostic BioSystems, Pleasanton, CA, USA). Immunostaining was performed in three independent biological experiments (*n* = 3). For each experimental condition, three representative fields were imaged, and the mean gray value was measured three times per image using the ImageJ software. The quantitative values represent the averages of these measurements. Image brightness and contrast were adjusted using BZ-X Analyzer exe version.1.3.0.3 (Keyence Corporation, Osaka, Japan), applying identical processing to all images without selective enhancement or removal of any specific features. Raw, unprocessed images are provided in Supplementary Fig. S7.

DAB signals were quantified using ImageJ/Fiji after the H-DAB color deconvolution. Five randomly selected non-overlapping fields were analyzed for each slide. The mean DAB intensity of the positively stained area was measured and normalized to the total tissue area (or cell number) in the field after subtracting the local background intensity. The average value of the five fields was used as a data point per slide.

### Statistical analysis

“Data analysis, including hierarchical cluster heatmaps, principal component analysis (PCA), two-way analysis of variance (ANOVA), and pathway analysis, was performed using MetaboAnalyst 6.0 (Wishart Research Group, Edmonton, AB, Canada) for metabolomic profiling and related statistical analyses. For pathway enrichment, we used the human Small Molecule Pathway Database (SMPDB) metabolite set library (99 curated human metabolic pathways). Pathways with a Benjamini–Hochberg false discovery rate (FDR) < 0.05 were considered significantly enriched. Basic data handling and descriptive statistics were performed using Microsoft Excel for Microsoft 365 (Microsoft Corporation, Redmond, WA, USA). A repeated-measures ANOVA was applied to assess group effects (GEs), time effects (TEs), and interaction effects (IEs), and two-tailed paired t-tests were used to compare values at individual time points. Data are expressed as mean ± standard deviation, and *p* < 0.05 was considered statistically significant.

The planned sample size was estimated a priori using G*Power version 3.1.9.7 (Heinrich-Heine-Universität Düsseldorf, Düsseldorf, Germany), which indicated that at least 20 samples per group (*n* ≥ 20) would be required to achieve adequate statistical power for the study. In practice, owing to experimental and biological limitations, most in vitro assays were performed with three biological replicates per group (*n* = 3), as this level of replication is widely adopted and practically achievable in cell-based experiments.

HG-control was used as the global reference. Statistical testing focused on (1) comparisons of each group versus HG-control and (2) comparisons between 3HB-treated and -untreated cells within the same medium (HG-control vs. HG-3HB and LG-control vs. LG-3HB). Other cross-condition contrasts (for example, HG-3HB vs. LG-3HB) were not considered primary outcomes and are not highlighted in the figures.

## Supplementary Information

Below is the link to the electronic supplementary material.

Supplementary Material 1

Supplementary Material 2

Supplementary Material 3

Supplementary Material 4

Supplementary Material 5

Supplementary Material 6

Supplementary Material 7

Supplementary Material 8

Supplementary Material 9

Supplementary Material 10

Supplementary Material 11

Supplementary Material 12

Supplementary Material 13

Supplementary Material 14

Supplementary Material 15

## Data Availability

The data supporting the findings of this study are available within the article and its supplementary materials. Additional data are available from the corresponding author upon reasonable request.
