# Supplementary figures and images for: Physiological medium and 3-hydroxybutyrate modulate autophagy-linked organelle remodeling in human external urethral sphincter myoblasts
Source: Sci Rep. 2026 Mar 16;16:13610. doi: 10.1038/s41598-026-43453-4 (PMC13121627; doi:10.1038/s41598-026-43453-4)

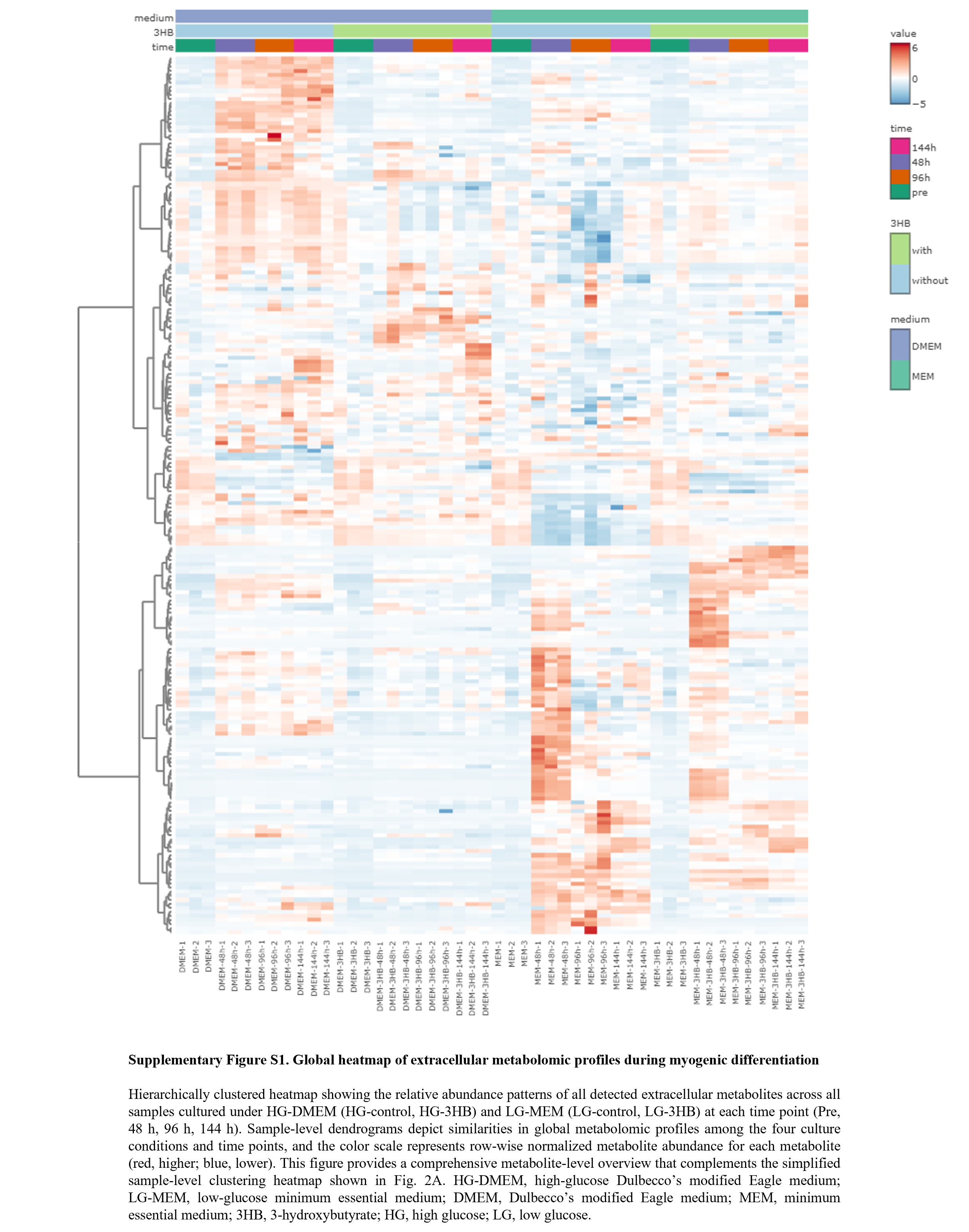

Supplement: Supplementary file 1 — Supplementary Material 1 [file 41598_2026_43453_MOESM1_ESM.tif]

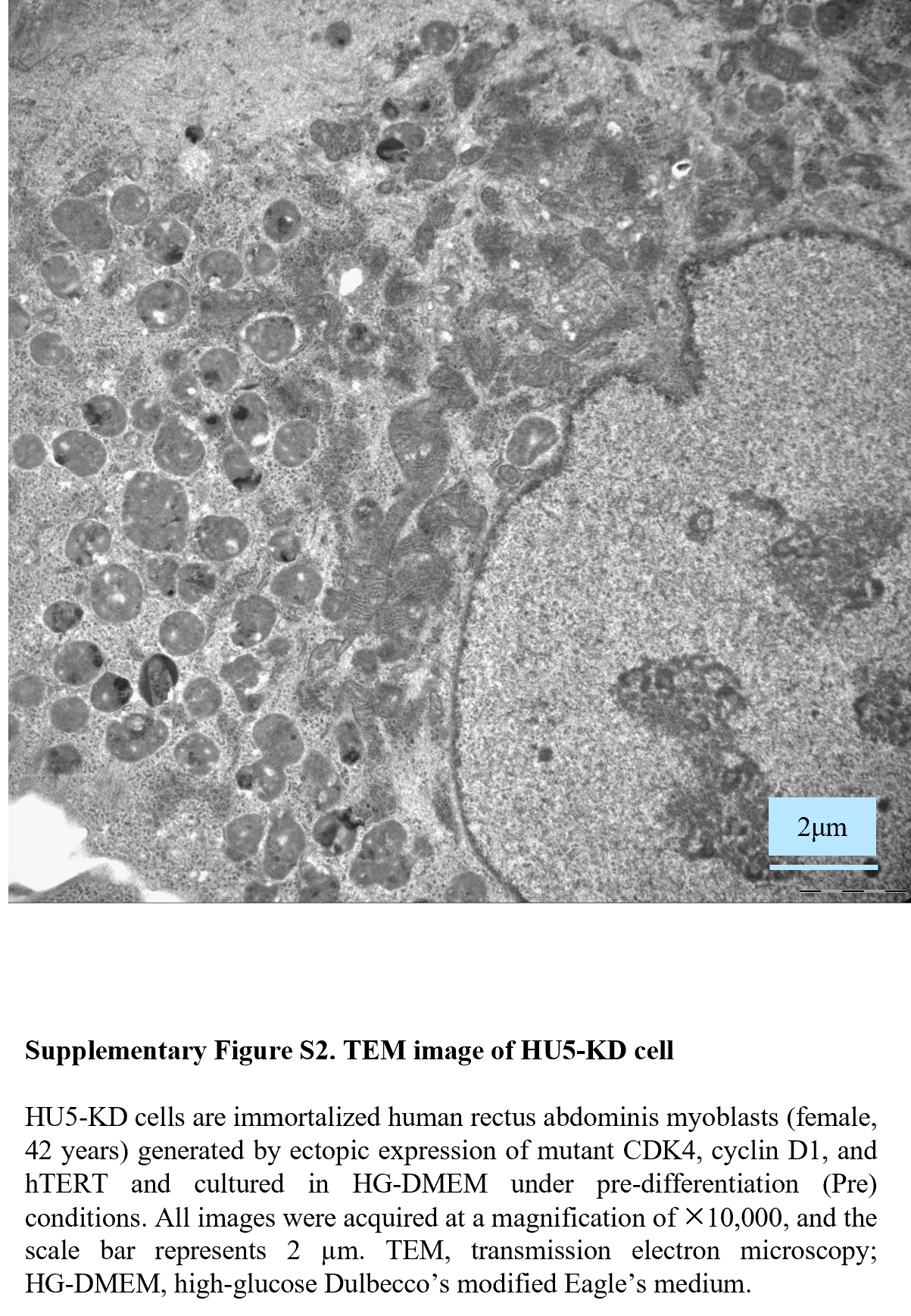

Supplement: Supplementary file 2 — Supplementary Material 2 [file 41598_2026_43453_MOESM2_ESM.tif]

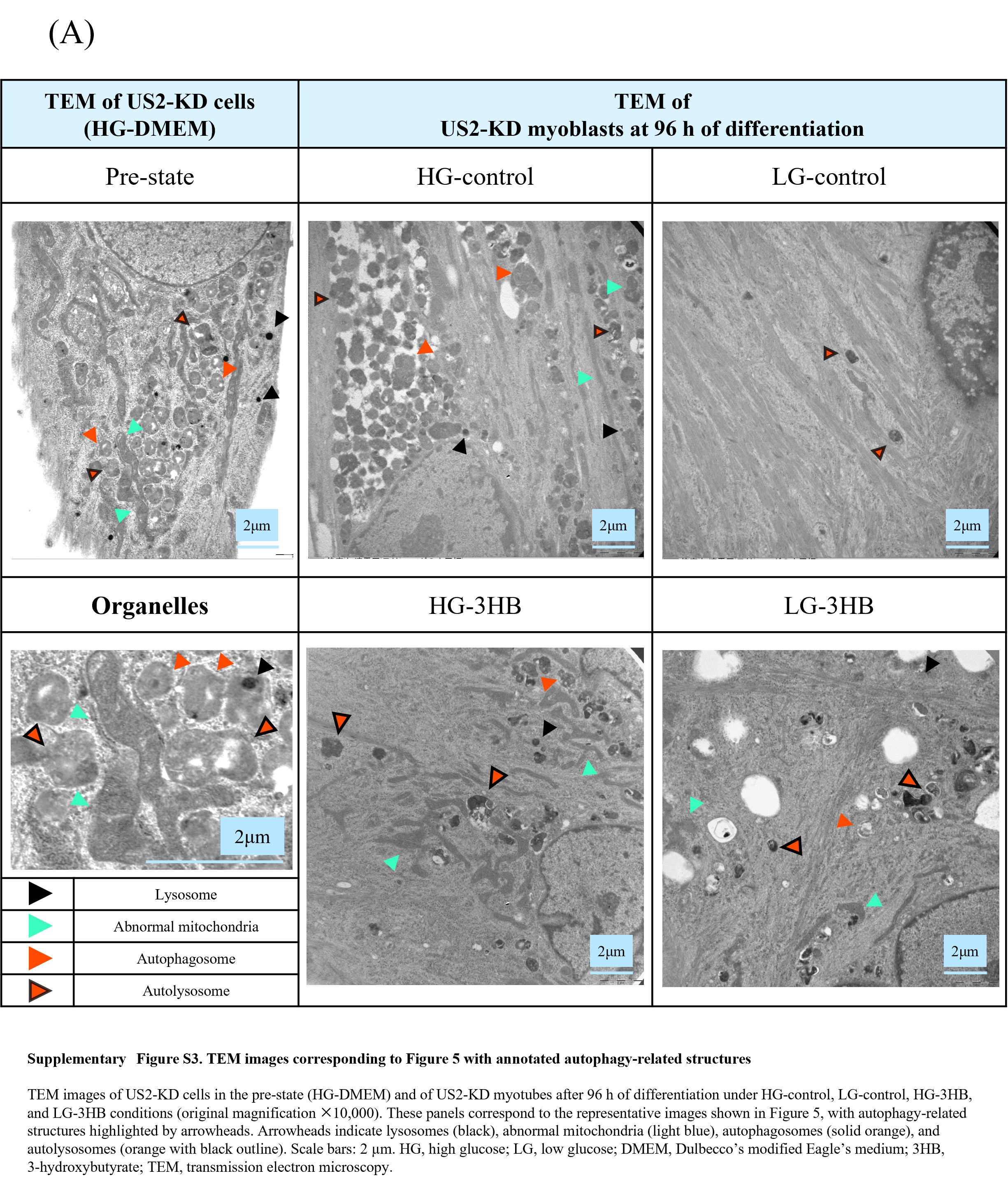

Supplement: Supplementary file 3 — Supplementary Material 3 [file 41598_2026_43453_MOESM3_ESM.tif]

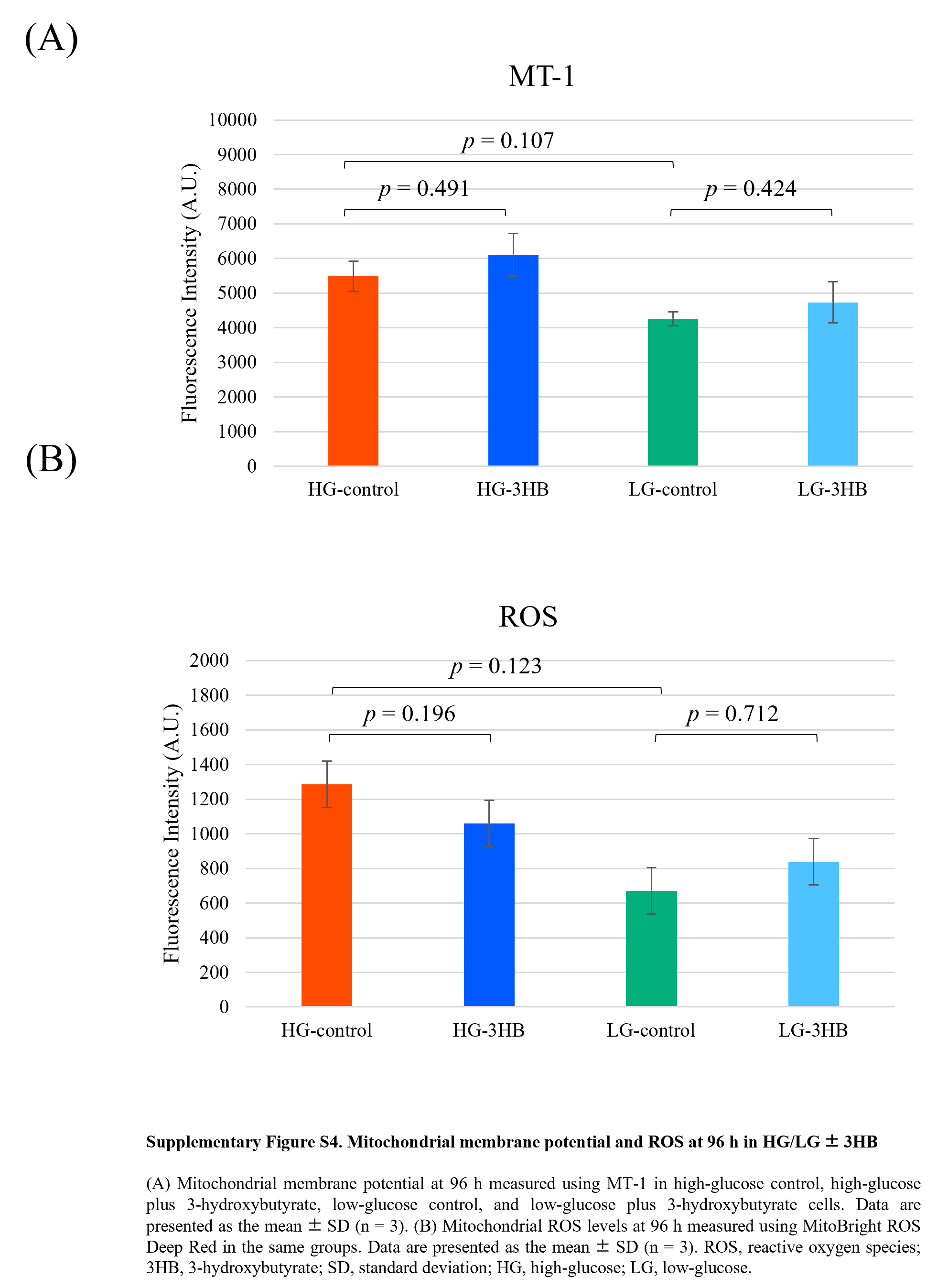

Supplement: Supplementary file 4 — Supplementary Material 4 [file 41598_2026_43453_MOESM4_ESM.tif]

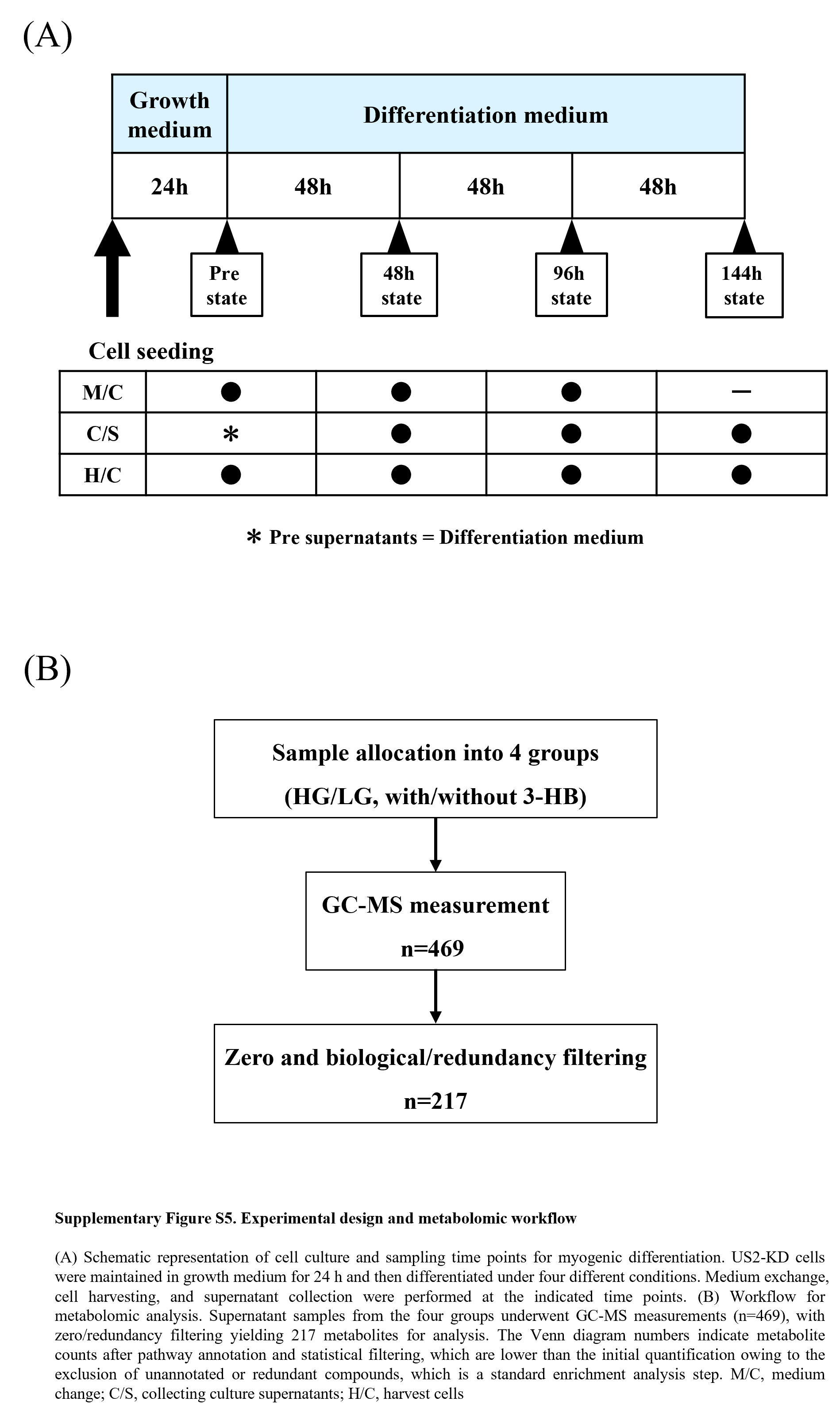

Supplement: Supplementary file 5 — Supplementary Material 5 [file 41598_2026_43453_MOESM5_ESM.tif]

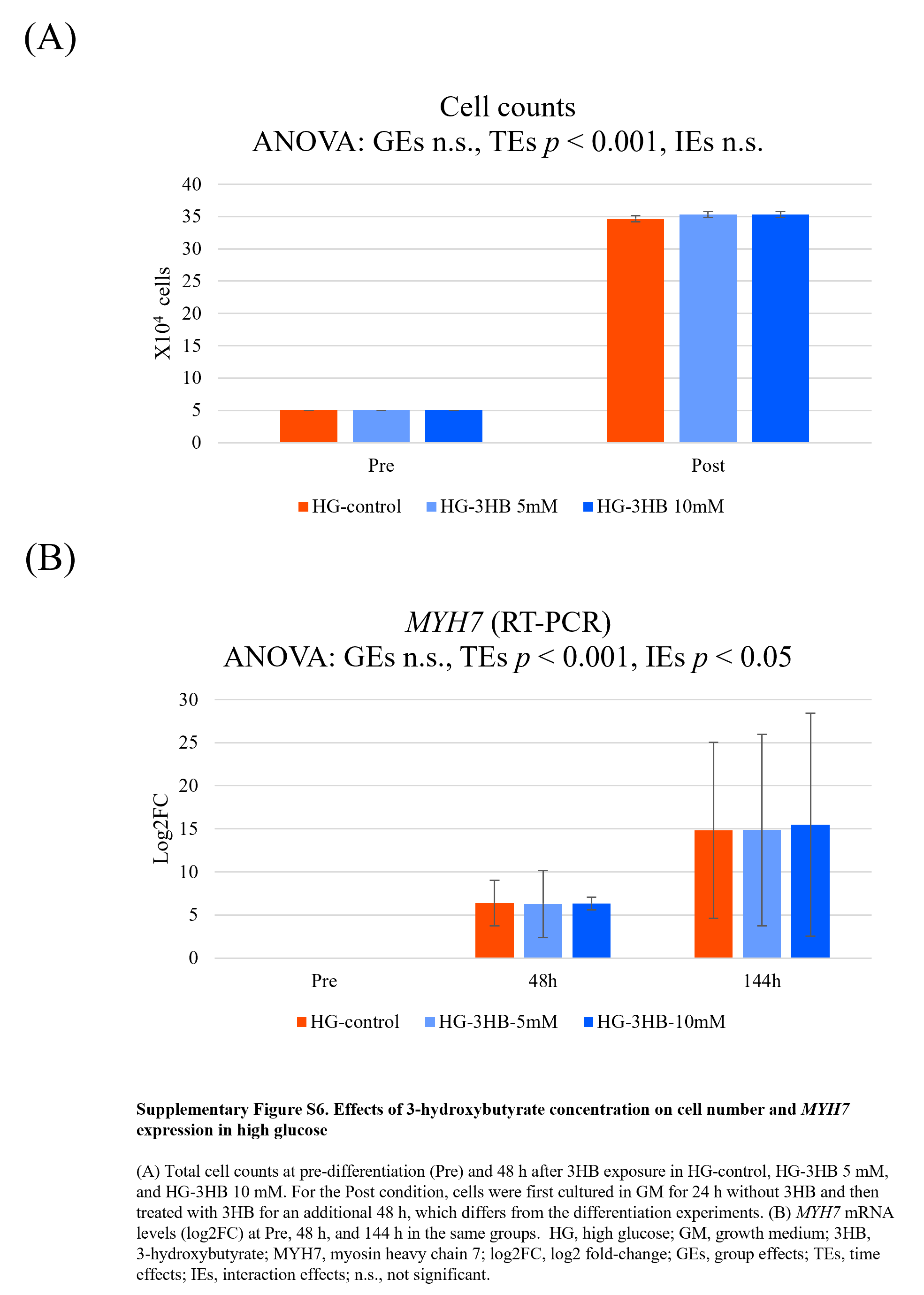

Supplement: Supplementary file 6 — Supplementary Material 6 [file 41598_2026_43453_MOESM6_ESM.tif]

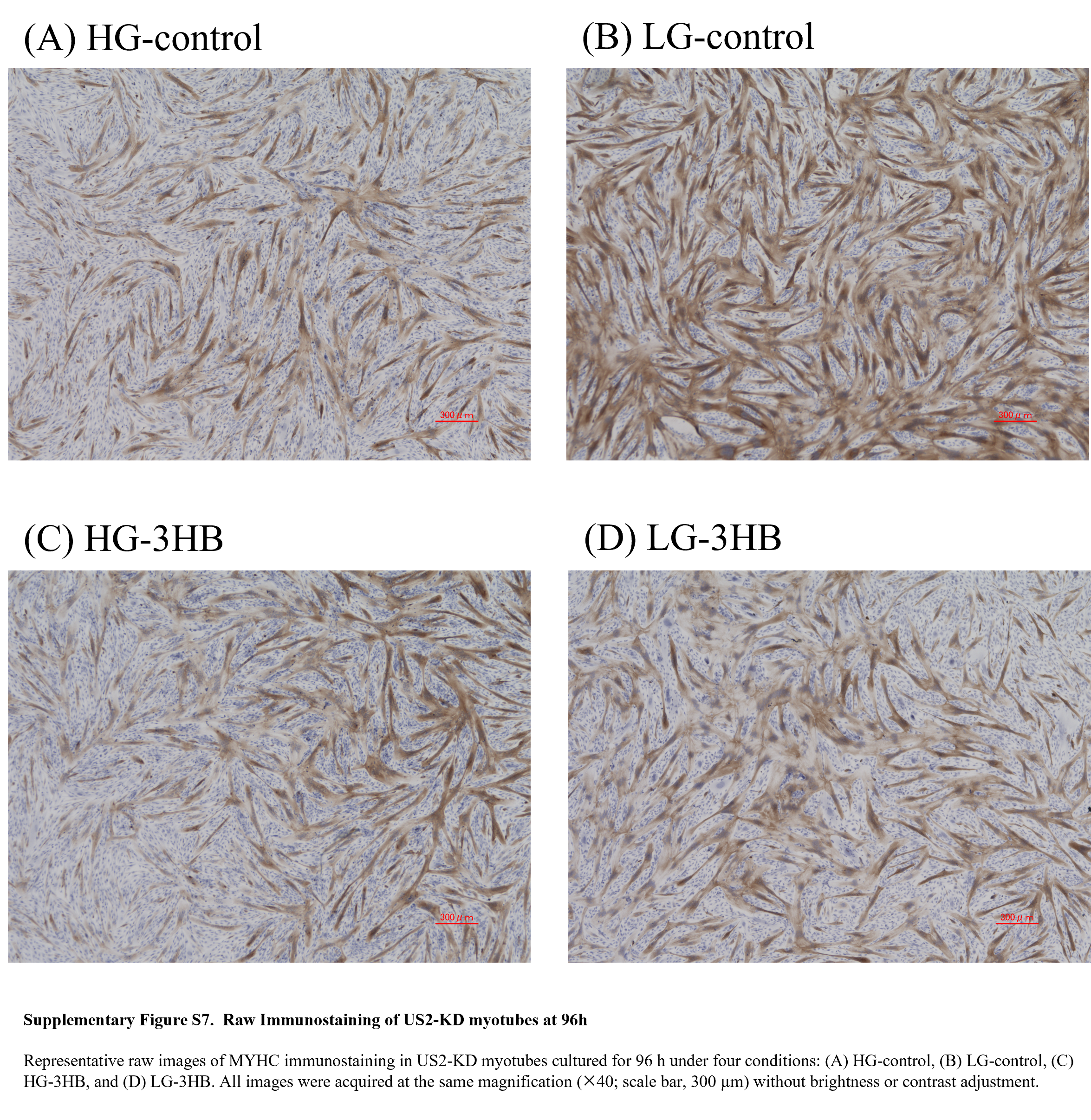

Supplement: Supplementary file 7 — Supplementary Material 7 [file 41598_2026_43453_MOESM7_ESM.tif]
